# MiR-630 inhibits proliferation by targeting CDC7 kinase, but maintains the apoptotic balance by targeting multiple modulators in human lung cancer A549 cells

**DOI:** 10.1038/cddis.2014.386

**Published:** 2014-09-25

**Authors:** J-X Cao, Y Lu, J-J Qi, G-S An, Z-B Mao, H-T Jia, S-Y Li, J-H Ni

**Affiliations:** 1 Department of Biochemistry and Molecular Biology, Peking University Health Science Center, Xue Yuan Road 38, Beijing 100191, People's Republic of China; 2 Department of Biochemistry and Molecular Biology, Capital Medical University, You An Men 8, Beijing 100069, People's Republic of China

## Abstract

MicroRNAome analyses have shown microRNA-630 (miR-630) to be involved in the regulation of apoptosis. However, its apoptotic role is still debated and its participation in DNA replication is unknown. Here, we demonstrate that miR-630 inhibits cell proliferation by targeting cell-cycle kinase 7 (CDC7) kinase, but maintains the apoptotic balance by targeting multiple activators of apoptosis under genotoxic stress. We identified a novel regulatory mechanism of *CDC7* gene expression, in which miR-630 downregulated CDC7 expression by recognizing and binding to four binding sites in CDC7 3'-UTR. We found that miR-630 was highly expressed in A549 and NIH3T3 cells where CDC7 was downregulated, but lower in H1299, MCF7, MDA-MB-231, HeLa and 2BS cells where CDC7 was upregulated. Furthermore, the induction of miR-630 occurred commonly in a variety of human cancer and immortalized cells in response to genotoxic agents. Importantly, downregulation of CDC7 by miR-630 was associated with cisplatin (CIS)-induced inhibitory proliferation in A549 cells. Mechanistically, miR-630 exerted its inhibitory proliferation by blocking CDC7-mediated initiation of DNA synthesis and by inducing G1 arrest, but maintains apoptotic balance under CIS exposure. On the one hand, miR-630 promoted apoptosis by downregulation of CDC7; on the other hand, it reduced apoptosis by downregulating several apoptotic modulators such as PARP3, DDIT4, EP300 and EP300 downstream effector p53, thereby maintaining the apoptotic balance. Our data indicate that miR-630 has a bimodal role in the regulation of apoptosis in response to DNA damage. Our data also support the notion that a certain mRNA can be targeted by several miRNAs, and in particular an miRNA may target a set of mRNAs. These data afford a comprehensive view of microRNA-dependent control of gene expression in the regulation of apoptosis under genotoxic stress.

Cell division cycle 7 (CDC7) is a conserved serine–threonine kinase essential for the initiation of DNA replication.^1,2^ Activation of CDC7 kinase requires its association with one of the regulatory proteins DBF4 and DRF1,^1, 2, 3^ which are cyclically expressed and reach a peak during the S phase.^4, 5, 6, 7^ CDC7 modulates S-phase checkpoint in DNA damage response (DDR)^8, 9, 10^ by attenuating checkpoint signaling and triggering DNA replication reinitiation.^11^ CDC7 may also phosphorylate claspin and activate ATR-CHK1 checkpoint pathway.^12^ CDC7 expression is very low or undetectable in normal tissues and cell lines but high in many human cancers and tumor cell lines.^13,14^ Silencing CDC7 in cancer cells impairs progression through the S phase, inducing p53-independent apoptosis, but does not influence normal cells.^15,16^ Therefore, CDC7 becomes an attractive target for cancer therapy.^17,18^

MicroRNAs (miRNAs) posttranscriptionally regulate gene expression. MiRNAs control ~30% protein-coding genes,^19^ and have roles in diverse biologic processes including proliferation, differentiation and apoptosis. As miRNAs may function as either tumor suppressor or oncogene, deregulation of miRNAs is closely related to tumorigenesis.^20, 21, 22, 23, 24, 25^ MiRNAs are involved in DDR. For instance, miRNA-34 family members are regulated by p53 in DDR and have roles in cell-cycle checkpoint and apoptosis.^26, 27, 28, 29^ Many miRNAs (miR-24, miR-16, miR-421 and miR-138) have roles in DNA damage and repair.^30, 31, 32, 33^ MiRNA-regulated DDR may have the potential to improve the efficacy of cancer therapy relying on induction of DNA damage. Further understanding of miRNA actions in regulating cell death and DNA damage under genotoxic stresses will provide insights into cancer surveillance and limiting tumor progression.

MicroRNA-630 (MiR-630) is induced by cisplatin (CIS) and 3-Cl-AHPC (an adamantyl retinoid-related molecule), and it causes apoptosis in certain types of cancer cells by targeting different molecules such as BCL2, BCL2L2 and IGF-1R.^34,35^ Moreover, miR-630 exerts cytoprotective effects in CIS-administered A549 cells, but rather behaves as a specific cell death modulator in oxaliplatin-exposed A549 and CIS-exposed H1650, H1975 and HCC827 cells.^36^ These observations indicate that the role of miR-630 in regulating apoptosis is not fully understood. Besides, direct targeting of a modulator involving in DNA replication by miRNA-630 is unknown. Here, we provide evidence that miR-630 downregulates CDC7 expression in A549 cells, thereby inhibiting CDC7-mediated DNA synthesis and contributing to CIS-induced inhibitory proliferation, but maintains the apoptotic balance by targeting multiple modulators.

## Results

### MiR-630 downregulates CDC7 by targeting CDC7 3'-UTR

**Figure 1:**
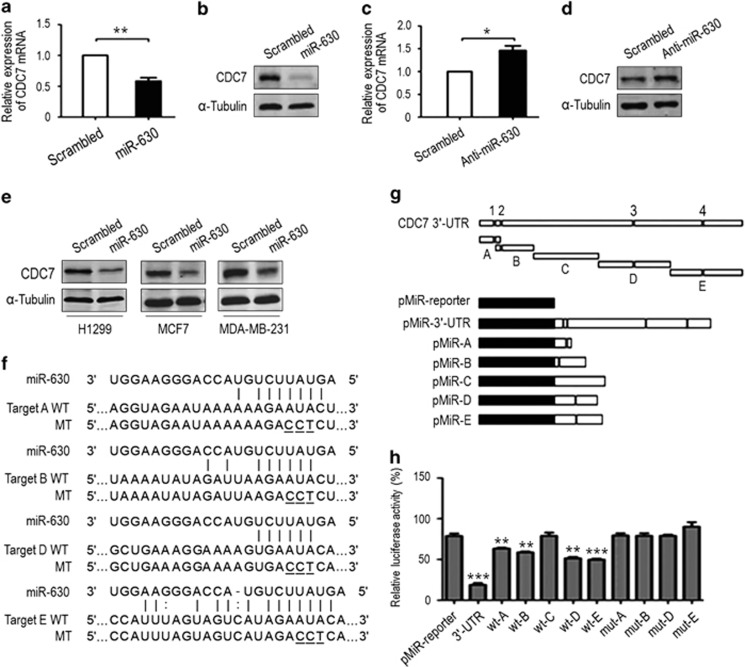
MiR-630 downregulates CDC7 expression by targeting CDC7 3'-UTR. A549 cells were transfected with miR-630 mimic or an inhibitor (50 nM) for 48 h. (**a**) RT-qPCR for CDC7 mRNA downregulation by miR-630. CDC7 mRNA was quantified by the 2^−ΔΔCt^ method, in which glyceraldehyde 3-phosphate dehydrogenase (GAPDH) was used as an internal control. The scrambled small interfering RNA (siRNA) was used as a negative control, under which condition the level of CDC7 mRNA was normalized to ‘1'. Data are presented as mean±S.D. (*n*=3). (**b**) Western blotting for CDC7 protein downregulation by miR-630. *α*-Tubulin was used as a loading control. (**c**) RT-qPCR and (**d**) western blotting for effects of miR-630 inhibitor on CDC7 mRNA and protein expression. Anti-scrambled siRNA was used as a control. Data are presented as mean±S.D. (*n*=3). (**e**) Western blotting for CDC7 protein in H1299, MCF7 and MDA-MB-231 cells transfected with miR-630 mimic for 48 h. (**f**) The predicted miR-630-binding sequences or mutated versions of CDC7 3'-UTR fragments ‘A', ‘B', ‘D' and ‘E' in (**g**). WT, wild type; MT, mutant (mutated bases are underlined). (**g**) Interpretation of luciferase reporter plasmids containing full-length CDC7 3'-UTR, fragments ‘A' to ‘E' or mutants (upper panel). The full-length 3'-UTR, truncates ‘A' to ‘E' and mutants in (**f**) were inserted into the pMIR-Report plasmid to generate pMIR-Report-PmiR-3'-UTR and its variations. ‘1' to ‘4' indicates the miR-630 binding sites. (**h**) Relative luciferase activities of the reporter plasmids in A549 cells. Data are presented as mean±S.D. (*n*=3). *P<0.05, **P<0.01 and ***P<0.001

Depletion of CDC7 induces apoptosis in cancer cells.^15,16^ MiR-630 may target BCL2, BCL2L2 and IGF-1R to induce apoptosis under genotoxic stresses.^34,35^ As an miRNA may have multiple targets,^14,37^ we speculated that miR-630-induced inhibitory proliferation and, perhaps, apoptosis might be linked to CDC7. To demonstrate this hypothesis, the potential targets of miR-630 were searched by TargetScan software (http://www.targetscan.org), and CDC7 was selected. To validate whether miR-630 could target CDC7, we performed real-time quantitative PCR (RT-qPCR) to check the transfection efficiency (Supplementary Figure S1) and CDC7 expression after transfection of miR-630 mimic and inhibitor into A549 (p53-wt) cells. RT-qPCR and western blotting revealed that compared with transfection of scrambled siRNA, transfection of miR-630 mimic caused marked decreases in CDC7 mRNA and protein (Figures 1a and b), whereas transfection of miR-630 inhibitor led to significant increases of CDC7 mRNA and protein (Figures 1c and d). CDC7 downregulation was also observed in miR-630 mimic-transfected H1299 (p53-null), MCF7 (p53-wt) and MDA-MB-231 (p53-mutant) cells (Figure 1e). These data indicate that miR-630 may target and inhibit CDC7 in a cell-type-independent manner.

To demonstrate further CDC7 as a target for miR-630, we performed luciferase reporter assays and mutation of the seed sequences. For this purpose, we analyzed the miR-630-binding sites within the sequences *in silico*, and found four predicted miR-630 sites in CDC7 3′-UTR. Full-length 3'-UTR, 3'-UTR fragments ‘A', ‘B', ‘D' and ‘E' (containing one putative miR-630 target site, respectively) and their mutants (Figure 1f), and fragment ‘C' (no miR-630 site) were inserted downstream of the pMiR-Reporter vector to generate a series of reporter constructs (Figure 1g). The resulting constructs were co-transfected with scrambled siRNA or miR-630 mimic into A549. Luciferase reporter assays showed that miR-630 mimic blocked the luciferase activity expressed by the construct containing full-length CDC7 3'-UTR compared with the activity of pMiR-Reporter (*P*<0.0001) (Figure 1h). Also, the constructs containing ‘A', ‘B', ‘D' or ‘E' fragment significantly reduced reporter enzyme activities (*P*=0.0098, *P*=0.0053, *P*=0.0086, *P*=0.0007, respectively) when the miR-630 mimic was transfected, whereas the ‘C' fragment-containing reporter did not lose its luciferase activity compared with control (pMiR-empty) vector. Moreover, the repression of luciferase activity was abolished when the putative miR-630 sites were mutated. These results indicate that miR-630 targets and downregulates CDC7 through direct binding to CDC7 3'-UTR. This conclusion was further evidenced by co-transfection of CDC7 3'-UTR-containing pEGFP-C3 vector with miR-630 mimic in GFP reporter assays (Supplementary Figure S2).

### MiR-630 is differentially expressed but commonly induced in cancer and immortalized cells upon DNA damage

**Figure 2:**
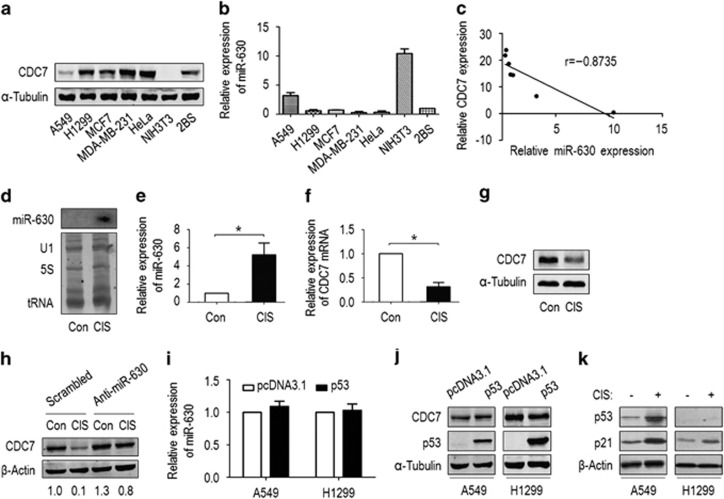
MiR-630 expression inversely correlates with CDC7 expression in a variety of cells. (**a**) Western blotting for CDC7 protein in A549, H1299, MCF7, MDA-MB-231, HeLa, NIH3T3 and 2BS cells. Cells were grown in culture for 48 h. Western blotting was performed with anti-CDC7 antibody. *α*-Tubulin was used as a loading control. (**b**) Stem-loop RT-PCR analysis of miR-630 in several cells. The expression levels of miR-630 were quantified by RT-qPCR and normalized to the levels of U6 snRNA. Data present mean±S.D. (*n*=3). (**c**) The correlation of CDC7 protein and miR-630 levels; *r*=−0.8735; *P*=0.0102. (**d**) Northern blotting for miR-630 expression in CIS-exposed A549. Cells were exposed to 100 *μ*M CIS for 36 h and Northern blotting was performed. U1, 5SRNA and tRNA were used as loading controls. Unexposed cells were used as control (Con). (**e**) RT-qPCR for miR-630 expression in CIS-exposed A549. Data are presented as mean±S.D. (*n*=3); *P=0.0347. (**f**) RT-qPCR and (**g**) western blotting for CDC7 mRNA and protein in CIS-exposed A549. Data are presented as mean±S.D. (*n*=3); *P=0.0171. (**h**) The effect of anti-miR-630 on CDC7 expression. A549 cells were transfected with miR-630 inhibitor (50 nM) for 48 h, and exposed to CIS for 36 h, followed by western blotting with anti-CDC7 antibody. The blots were screened/quantified and normalized against *β*-actin level. The value obtained from scrambled small interfering RNA (siRNA)/CIS-unexposed cells was designated as ‘1' (lane 1, bottom). (**i** and **j**) The effects of p53 transfection on miR-630 and CDC7 expression. A549 (p53-wild type) and H1299 (p53-null) cells were transfected with pcDNA3.1-p53 (pcDNA3.1 as control) for 48 h, followed by RT-qPCR for miR-630 (**i**) and western blotting for CDC7 and p53 (**j**). Data are presented as mean±S.D. (*n*=3) in (**i**), and *α*-tubulin was used as a loading control in (**j**). (**k**) P53 status in A549 and H1299. Cells were grown and exposed or unexposed to CIS for 36 h, followed by western blotting for p53 and p21 expression, and *β*-actin was used as a loading control

To study the correlation of CDC7 and miR-630 in cells, CDC7 and miR-630 expression in several cell lines was examined. CDC7 was expressed relatively lower or undetectable in A549 and NIH3T3, but high in H1299, MCF7, MDA-MB-231, HeLa and 2BS cells (Figure 2a). Contrarily, miR-630 was expressed very high in A549 and NIH3T3, but lower in H1299, MCF7, MDA-MB-231, HeLa and 2BS (Figure 2b). The scatter plot for CDC7 *versus* miR-630 revealed an inverse correlation of CDC7 and miR-630 in these cells (*r*=−0.8735, *P*=0.0102) (Figure 2c). These data indicate again that miR-630 acts as a modulator of CDC7. These data also suggest that miR-630 as well as CDC7 is differentially expressed in a variety of cancer and immortalized cells.

To investigate miR-630 induction by genotoxic stress, we exposed A549 cells to CIS for 36 h. Northern blotting showed that miR-630 was upregulated under CIS exposure (Figure 2d). RT-qPCR revealed that CIS exposure increased the level of miR-630 by ~5.2-folds (Figure 2e). Whereas the levels of CDC7 mRNA and protein were markedly decreased in CIS-exposed A549 compared with unexposed (control) cells (Figures 2f and g). To demonstrate further CDC7 downregulation by miR-630 under CIS exposure, anti-miR-630 was transfected into A549, and CDC7 was tested 36 h after CIS exposure. CDC7 downregulation was markedly abrogated by anti-miR-630 compared with scrambled siRNA (Figure 2h). The opposite expression of miR-630 and CDC7 was also detectable in camptothecin (CPT)- and CdCl_2_-exposed A549 (Supplementary Figure S3). Furthermore, miR-630 induction was observed in H1299, MCF7, MDA-MB-231 and 2BS cells exposed to CIS (data not shown). To clarify whether miR-630 induction was associated with p53, pcDNA3.1-p53 expression vector was transfected into A549 and H1299, followed by examining miR-630 and CDC7. Neither miR-630 nor CDC7 was affected in both cells (Figures 2i and j), regardless of the status of p53 and its target p21 before and after exposure (Figure 2k). These data indicate that miR-630 induction by CIS may not be regulated by p53, and is a common event in human cancer and immortalized cells under genotoxic stresses.

### CDC7 downregulation contributes to CIS-induced inhibitory proliferation and apoptosis in A549 cells

**Figure 3:**
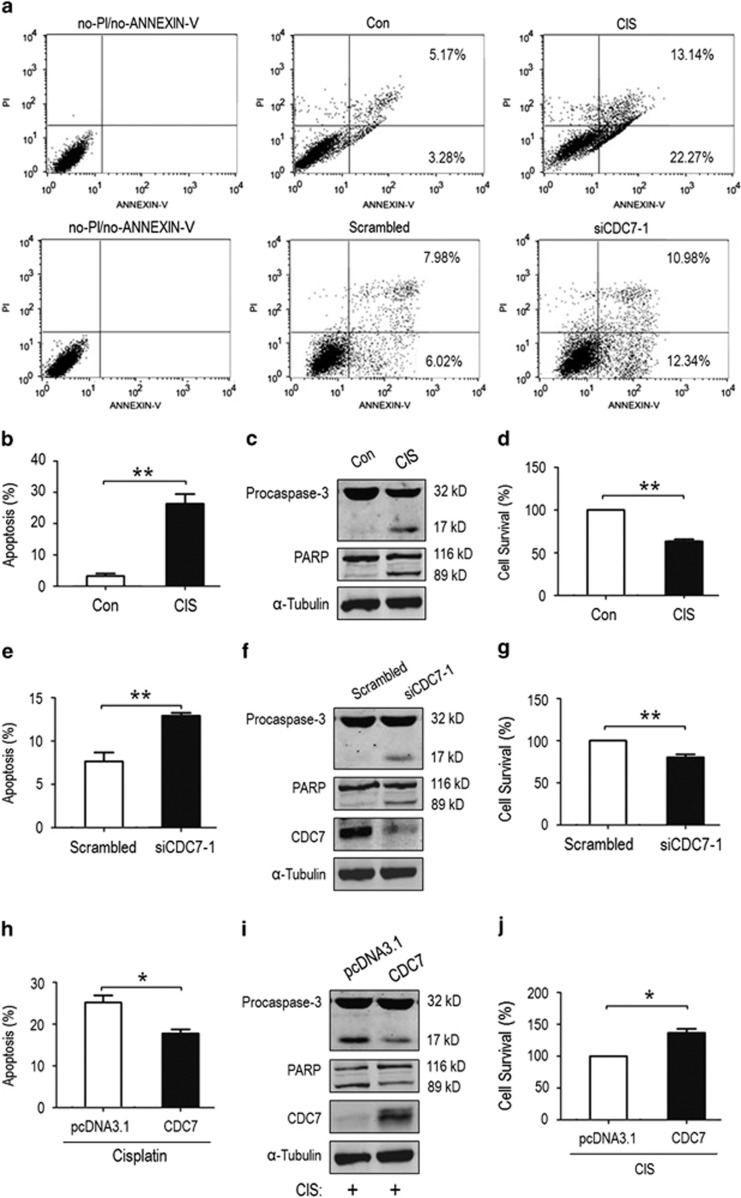
Downregulated CDC7 contributes to CIS-induced inhibitory proliferation and apoptosis. (**a**) Representative of flow cytometry for CIS-induced apoptosis. A549 cells were exposed to CIS for 36 h (upper panel) or transfected with CDC7 small interfering RNA (siRNA) (siCDC7-1) for 48 h to silence CDC7 (lower panel). Apoptosis was analyzed by Annexin V/PI double staining and flow cytometry. The cells in the bottom-right quadrant were stained by Annexin V (early apoptosis); top-right quadrant, cells stained by PI and Annexin V (late apoptosis/necrosis). (**b**) Apoptosis in CIS-exposed A549 cells in (**a**) experiments (upper panel). Data are presented as mean±S.D. (*n*=3). (**c**) Procapase-3 activation and poly (ADP-ribose) polymerase (PARP) cleavage in CIS-exposed A549 cells. The activated caspase-3 (p17) and cleaved PARP (p89) were examined by western blotting using a specific antibody. (**d**) MTS assay showed the survival of A549 cells exposed to CIS. Data are presented as mean±S.D. (*n*=3). (**e**) Induction of apoptosis by silencing CDC7 in A549 cells. For dot plots in flow cytometry, see (**a**) lower panel. Data are presented as mean±S.D. (*n*=3). (**f**) Western blotting for procaspase-3, PARP and CDC7 in CDC7-silenced A549. (**g**) MTS assay showed the survival of CDC7-silenced A549 cells. Data are presented as mean±S.D. (*n*=3). (**h** and **i**) Reduction of apoptosis by CDC7 overexpression. A549 cells were transfected with CDC7 expression plasmid for 48 h, and exposed to CIS for 36 h, followed by flow cytometry for apoptosis (**h**) and western blotting for procaspase-3 and PARP, and CDC7 (**i**). Data are presented as mean±S.D. (*n*=3). (**j**) MTS assay showed the survival of CDC7-overexpressed A549 cells. Data are presented as mean±S.D. (*n*=3). For a second CDC7 RNAi (siCDC7-2) experiments and dot plots in (**h**) see Supplementary Figures S4 and S5. *P<0.05 and **P<0.01

To examine the roles of downregulation of CDC7 by genotoxic agent in cell proliferation and apoptosis, A549 cells were exposed to CIS for 36 h, and apoptosis was determined by Annexin V/PI double staining and flow cytometry (Figure 3a, upper panel). The early apoptosis induced by CIS was increased by ~23% (*P*=0.0033) (Figure 3b). CIS-induced apoptosis was also confirmed by the activation of procaspase-3 that was cleaved to generate a 17 kDa activated product, which in turn led to cleavage of its substrate PARP (Figure 3c). Furthermore, CIS exposure reduced cell survival by 36.7% in non-radioactive cell proliferation assay (MTS) (*P*=0.0049) (Figure 3d). To demonstrate whether CDC7 was associated with CIS-induced apoptosis, CDC7 was silenced in A549 by CDC7 siRNA (siCDC7-1). Silencing CDC7 increased early apoptosis from 7.7% (scrambled siRNA) to 12.9% (*P*=0.0086) (Figures 3a and e, lower panel), followed by procaspase-3 activation (Figure 3f), whereas cell survival decreased by 19.8% compared with control (Figure 3g). The effects of silencing CDC7 on apoptosis and cell survival were further demonstrated by a second siRNA (siCDC7-2) (Supplementary Figure S4). Taken together, CDC7 downregulation contributes to CIS-induced inhibitory proliferation and apoptosis.

To further test the role of CDC7 in CIS-induced inhibitory proliferation and apoptosis, CDC7 was overexpressed in A549, followed by CIS exposure. Compared with pcDNA3.1-empty vector transfection, overexpressing CDC7 reduced CIS-induced apoptotic cells from 25.8 to 17.7% (*P*=0.0194) (Figure 3h and Supplementary Figure S5). Consistently, the activated product of procaspase-3 was reduced compared with CIS-exposed A549 without overexpressing CDC7 (Figure 3i). Unsurprisingly, overexpressing CDC7 increased cell survival even in the presence of CIS compared with control (Figure 3j). These data indicate that CDC7 expression increases cell resistance to CIS-induced apoptosis.

### MiR-630 inhibits proliferation by blocking CDC7-mediated DNA synthesis

**Figure 4:**
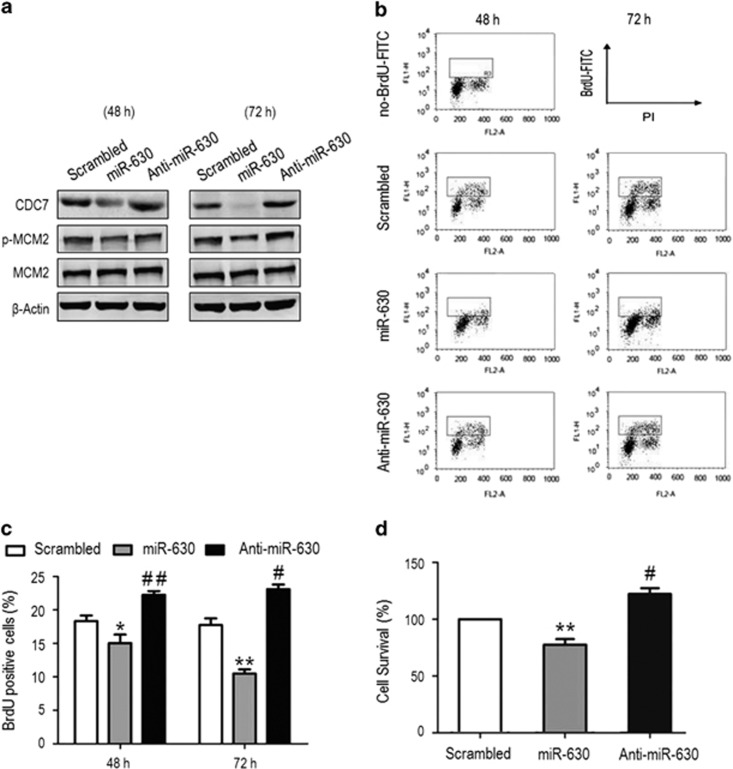
MiR-630 inhibits cell proliferation by inhibiting CDC7-mediated DNA synthesis. A549 cells were transfected with scrambled small interfering RNA (siRNA) (control), miR-630 mimic and miR-630 inhibitor for 48 and 72 h. (**a**) Western blotting for CDC7, MCM2 and phospho-MCM2. *β*-Actin was used as a loading control. (**b**) Flow cytometric analysis of BrdU-positive cells. After 48 and 72 h transfection, cells were labeled with 50 *μ*M BrdU for 1 h before collection. Samples were stained with anti-BrdU FITC antibody and PI, and analyzed by flow cytometry. BrdU-positive cells were included in the gate region. (**c**) Relative amount of BrdU-positive cells in the gate region compared with total cells in (**b**) experiments. Data are presented as mean±S.D. (*n*=3); 48 h: *P=0.0396, ^##^*P*=0.0054; 72 h: **P=0.0037, ^#^*P*=0.0171. (**d**) MTS assay showed the survival of A549 cells transfected with miR-630 mimic or inhibitor for 48 h. Data are presented as mean±S.D. (*n*=3); **P=0.0026 and ^#^*P*=0.0223

MCM2 phosphorylation by CDC7 promotes binding of CDC45 to the pre-RC during replisome assembly.^3,7^ To study the effects of CDC7 downregulation by miR-630 on DNA replication initiation and progression, miR-630 mimic or inhibitor was transfected into A549. After 48 and 72 h transfection, CDC7 and phosphorylated MCM2 were downregulated by miR-630 mimic compared with miR-630 inhibitor and scrambled siRNA (Figure 4a). To analyze DNA synthesis, transfected cells were labeled with bromo-deoxyuridine (BrdU) and double-stained with anti-BrdU antibody and propidium iodide (PI), followed by flow cytometry assays (Figure 4b). Compared with scrambled siRNA-transfected cells, the number of BrdU-positive cells increased 3.9% and 4.8% at 48 and 72 h in miR-630 inhibitor-transfected cells (*P*=0.0054; *P*=0.0171), but reduced 3.3% and 7.8% in miR-630 mimic-transfected cells (*P*=0.0396; *P*=0.0037), respectively (Figure 4c). Consistently, miR-630 mimic reduced cell survival by ~23% after 48 h transfection, whereas miR-630 inhibitor increased survival by 22.2% (Figure 4d). These results suggest that inhibitory proliferation of A549 cells by miR-630 is attributed to the inhibition of CDC7-mediated DNA synthesis.

### MiR-630 maintains apoptotic balance in A549 cells because of its multiple target roles

**Figure 5:**
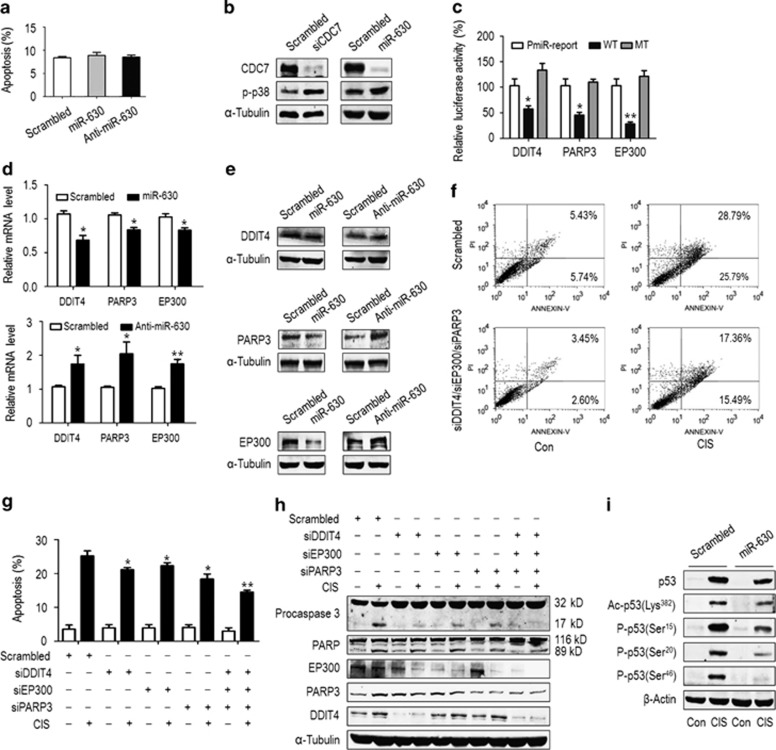
MiR-630 maintains the apoptotic balance by targeting multiple apoptotic regulators. (**a**) Annexin V/PI assay for apoptosis in A549 cells. Cells were transfected with scrambled small interfering RNA (siRNA), miR-630 mimic or inhibitor for 48 h. After double staining with Annexin V/PI, flow cytometry were performed. Data are presented mean±S.D. (*n*=5). For flow cytometry dot plots see Supplementary Figure S6. (**b**) Activation of p38 kinase by miR-630 transfection or silencing CDC7. A549 cells were transfected with scrambled siRNA, miR-630 mimic or CDC7 siRNA (siCDC7-1) for 48 h, followed by western blotting for CDC7 and p-p38 expression with specific antibodies. *α*-Tubulin was used as a loading control. (**c**) Relative luciferase activities of the reporter plasmids. Luciferase reporter constructs were co-transfected with scrambled siRNA or miR-630 mimic into A549. Luciferase reporter activities were assayed 48 h after transfection and normalized to scrambled siRNA. Data are presented as mean±S.D. (*n*=3). (**d**) The effects of miR-630 on DDIT4, PARP3 and EP300 mRNA expression. A549 cells were transfected with scrambled siRNA, miR-630 mimic (upper panel) and inhibitor (lower panel) for 48 h, followed by RT-qPCR for DDIT4, PARP3 and EP300 mRNA. Glyceraldehyde 3-phosphate dehydrogenase (GAPDH) was an internal control. Data are presented as mean±S.D. (*n*=3). (**e**) The effects of miR-630 on DDIT4, PARP3 and EP300 protein expression. Transfection of A549 was described in (**d**), and PARP3, EP300 and DDIT4 proteins were analyzed by western blotting. *α*-Tubulin was used as a loading control. (**f**) and (**g**) Reduction of apoptosis by silencing DDIT4, PARP3 and EP300. DDIT4, PARP3 and EP300 were individually or jointly silenced by transfection of specific siRNA oligonucleotides into A549 cells, and apoptosis was examined 48 h after transfection. Data are presented as mean±S.D. (*n*=3). (**f**) A representative of flow cytometry for apoptosis induced by combined silencing of DDIT4, PARP3 and EP300. For all dot plots in (**g**) see Supplementary Figure S8. (**h**) Western blotting for activated caspase-3 (p17), PARP (p89) and silenced DDIT4, PARP3 and EP300 in (**g**) experiments. (**i**) Downregulation of CIS-induced p53 and its modifications by miR-630. A549 cells were transfected with scrambled siRNA or miR-630 mimic for 48 h, and exposed to CIS for additional 36 h. The acylation of p53 at Lys382 and phosphorylation of p53 at Ser15, Ser20 and Ser46 were examined by western blotting. *β*-Actin was used as a loading control. *P<0.05 and **P<0.01

To determine the effects of miR-630-downregulated CDC7 on CIS-induced apoptosis, miR-630 mimic or inhibitor was transfected into A549, followed by the detection of apoptosis. Unexpectedly, in Annexin V/PI assays we could not detect any apoptosis in miR-630 mimic-transfected A549 compared with scrambled siRNA and inhibitor transfection (Figure 5a and Supplementary Figure S6), although miR-630 mimic as well as CDC7 siRNA markedly repressed CDC7 expression (Figure 5b). Consistently, procaspase-3 activation was undetectable (data not shown) in miR-630 mimic-transfected A549, indicating that miR-630 overexpression failed to induce apoptosis. As apoptosis induced by CDC7 depletion in cancer cells is associated with ATR-activated p38 kinase,^16^ we examined phosphorylation/activation of p38 kinase in CDC7-silenced A549 cells, where phosphor-p38 was upregulated (Figure 5b, left panel). Surprisingly, the activated kinase was also detectable in miR-630 mimic-transfected A549 (right panel). These results suggest that the p38 kinase-related apoptotic pathway is still operative in miR-630-expressed or CDC7-silenced A549. Thus, the functional significance of miR-630 induction in CIS-induced apoptosis remains to be established.

An mRNA can be regulated by several miRNAs and an miRNA may target multiple mRNAs.^37^ We thus speculated that miR-630 might have a bimodal role in the regulation of apoptosis because of its multiple target roles, thereby balancing apoptosis against antiapoptosis. We searched the potential targets of miR-630 on cell apoptosis and found that besides CDC7, DDIT4, PARP3 and EP300 3'-UTRs, all contain one miR-630-binding site (Supplementary Figure S7), indicating that they may function as miR-630 targets in the regulation of apoptosis. Indeed, these molecules have been shown to be involved in apoptotic process in various cancer cells.^34,38, 39, 40^ To demonstrate DDIT4, PARP3 and EP300 were targets for miR-630, the full-length 3'-UTRs and their mutants (Supplementary Figure S7) were inserted downstream of the pMiR-Reporter vector to generate wild-type and mutant reporters, respectively. The resulting constructs were co-transfected with scrambled siRNA or miR-630 mimic into A549. Luciferase reporter assays showed that miR-630 mimic inhibited 44.7% (*P*=0.0362), 56.3% (*P*=0.0165) and 72.8% (*P*=0.0059) activities of DDIT4-Luc, PARP3-Luc and EP300-Luc compared with pMiR-Reporter, respectively, but could not affect their mutant activities (Figure 5c). Furthermore, miR-630 mimic significantly decreased the expression of DDIT4, PARP3 and EP300 mRNA (Figure 5d, upper panel) and of protein (Figure 5e, left panel) in A549 cells, whereas anti-miR-630 increased their mRNA (Figure 5d, lower panel) and protein (Figure 5e, right panel) compared with scrambled siRNA, indicating DDIT4, PARP3 and EP300 as targets for miR-630. Next, we performed individual and combined silencing of three molecules in A549 by specific siRNA oligonucleotides^41,42^ (also see http: //www.pnas.org/cgi/content/short/1016574108) to examine the effects of targeting DDIT4, PARP3 and EP300 by miR-630 on CIS-induced apoptosis. Compared with scrambled siRNA-transfected A549, where CIS exposure induced ~25.2% early apoptosis, silencing DDIT4, PARP3 or EP300 reduced 4.1% (*P*=0.0435), 2.9% (*P*=0.0476) and 6.8% (*P*=0.0363) apoptosis, respectively; in particular, combined silencing of them reduced 10.7% apoptosis (*P*=0.0085) (Figures 5f and g and Supplementary Figure S8). Consistently, the inhibition of procaspase-3 and PARP cleavage was detected (Figure 5h). Taken together, targeting of DDIT4, PARP3 and EP300 by miR-630 reduces apoptosis. EP300 promotes apoptosis through acetylating p53.^39,40^ We detected p53 acetylation in miR-630-overexpressed A549 under CIS exposure. The levels of Lys382-acetylated p53 and total p53 were increased in scrambled siRNA-transfected cells exposed to CIS, whereas miR-630 transfection markedly reduced Lys382-acetylated p53, followed by total p53 declination (Figure 5i). Notably, miR-630 also inhibited Ser15-, Ser20- and Ser46-p53 phosphorylation in CIS-exposed and miR-630-expressed A549.

Taken together, miR-630 not only induces apoptosis by targeting CDC7 but also protect the cell from apoptosis by direct and indirect downregulation of DDIT4, PARP3, EP300 and p53. Alternatively, miR-630 may exert a bimodal role in the regulation of apoptosis by targeting multiple modulators of apoptosis, thereby maintaining the apoptotic balance.

### MiR-630 induction is associated with CIS-induced G1 arrest

**Figure 6:**
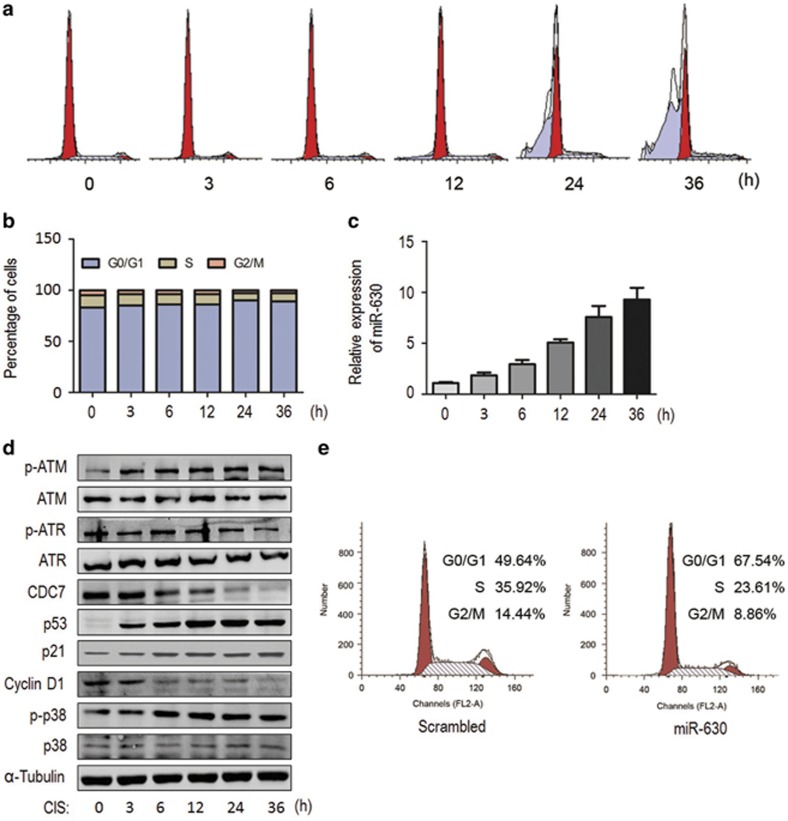
Induction of miR-630 is associated with CIS-induced G1 arrest. A549 cells were synchronized at G0/G1 phase by serum starvation for 48 h, re-feeding with 20% FBS and treated with CIS for 0, 3, 6, 12, 24 and 36 h, respectively. (**a**) A representative of flow cytometry showing CIS induced G1 arrest. (**b**) Histograms showing the populations of the cell-cycle in (**a**) experiments. Cells (2 × l0^5^) were fixed and stained with PI, and analyzed by FACScan. Data present mean from two independent experiments. (**c**) The expression of miR-630 in (**a**) experiments. MiR-630 was determined by RT-qPCR and U6 was used as internal control. Data present mean±S.D. (*n*=3). (**d**) Western blotting for ATM/p-ATM, ATR/p-ATR, CDC7, p53, p21, cyclin D1, p38/p-p38 in (**a**) experiments. *α*-Tubulin as loading control. (**e**) G1 arrest in miR-630 mimic-transfected A549 cells. Cells were transfected with miR-630 mimic (scrambled small interfering RNA (siRNA) as control) for 48 h, followed by analysis of the cell-cycle (for CDC7 and p-p38 expression see Figure 5b, right panel)

To investigate the effects of CIS-induced miR-630 on cell cycle, A549 cells were synchronized at G0/G1-phase by serum starvation and released into the cell cycle by adding 20% fetal calf serum and exposed to CIS for given hours, followed by cell-cycle analysis. G1 arrest was observed immediately after CIS exposure and sustained until at least 36 h (Figures 6a and b). The majority of G1 population with a sub-G1 peak appeared 24 h after exposure (Figure 6a). Meanwhile, miR-630 was upregulated within 3–36 h after exposure (Figure 6c). Contrarily, CDC7 was markedly downregulated after 6 h exposure (Figure 6d). Supporting G1 arrest, CIS exposure increased p-ATM, p53 and p21 expression, whereas cyclin D1 was downregulated after 6 h exposure. Phospho-ATR was consistently detectable, although its levels were not increased. Moreover, phospho-p38 was increased (Figure 6d). These data indicate that CIS exposure may induce G1 arrest and apoptosis in A549. It seems likely that CIS-induced G1 arrest is attributed to the activation of ATM/ATR-p53-p21 signaling pathway, and CIS-induced apoptosis is linked to the activation of p53 and p38 kinase.

To demonstrate whether miR-630 induction was associated with CIS-induced G1 arrest, miR-630 mimic or scrambled siRNA was transfected into A549 cells, and the cell cycle was analyzed 48 h after transfection. MiR-630 overexpression significantly increased G1 sub-population (67%) compared with scrambled siRNA-transfected cells (49% G1 sub-population) (Figure 6e), indicating that miR-630 induction is associated with CIS-induced G1 arrest but not apoptosis.

## Discussion

In this study, we demonstrate that miR-630 as a DDR component is induced upon CIS exposure, leading to subsequent modulation of several targets involved in DNA replication and apoptosis. First, we identified a novel regulatory mechanism of *CDC7* gene, whose expression is suppressed by miR-630 by targeting CDC7 3'-UTR. Second, miR-630-suppressed CDC7 inhibits CDC7-mediated initiation of DNA synthesis and induces G1 arrest, contributing to CIS-inhibited cell proliferation. Third, miR-630 may exert a bimodal role in the regulation of apoptosis by targeting multiple modulators of apoptosis, therebymaintaining the apoptotic balance.

CDC7 is frequently downregulated in response to genotoxic stresses, resulting in the activation of S-phase checkpoint signal.^8,9,11,15,16^ Overexpression of CR/periphilin may also downregulate CDC7 and induce S-phase arrest.^43^ Recently, CDC7 was shown to be downregulated by miR-29a under BPDE-induced DNA damage.^44^ We found that miR-630 downregulated CDC7 by targeting its 3'-UTR (Figure 1). MiR-630 induction and CDC7 suppression were detectable in A549 exposed to CIS (Figures 2d–h), CPT and CdCl_2_ (Supplementary Figure S3). We also observed miR-630 induction in H1299, MCF7, MDA-MB-231, HeLa and 2BS cells under CIS exposure (data not shown). The inverse correlation of miR-630 and CDC7 expression occurred in several cancer and immortalized cells even in the absence of DNA damage agents (Figures 2a–c). We conclude that miR-630 acts as a modulator of CDC7. Our data also suggest that the regulatory mechanism of CDC7 by miR-630 is operative in cancer and immortalized cells, in particular when exposed to DNA-damaging agents. MicroRNAome studies show that several miRNA species are deregulated in cancer cells during CIS-induced DNA damage, in which miR-630 is upregulated.^34,36^ Taking the previous findings^34,36^ and our data, miR-630 induction is a DDR component. MiR-29a is not included in the deregulated miRNA species under CIS exposure.^34,36^ It seems that targeting CDC7 by miR-630 or miR-29a depends upon cell contexts and genotoxic agents.

DNA damage may regulate miRNA expression at the transcriptional level, in which transcription factors c-Myc,^45^ p53^46^ and E2F1^47, 48, 49^ have roles. DNA damage may also regulate miRNA expression by modulating miRNA processing and maturation.^50^ We found that p53 overexpression could not induce miR-630 and suppress CDC7 in A549 and H1299 cells (Figures 2i and j). Also, we observed miR-630 induction in p53-deficient H1299, MDA-MB-231 and HeLa cells under CIS exposure (data not shown). Our data indicate that miR-630 induction may not be associated with p53. A recent study showed that regulation of the DICER1 promoter by ATM-phosphorylated delta-DNp63a was implicated in CIS-induced alteration in microRNAome (including miR-630).^34^ We reported recently that CIS-induced miR-630 was attributed to the promotion of pri-miR-630 processing by E2F1-regulated DROSHA.^51^ However, activation of the *miR-630* gene by DNA damage is unknown.

CDC7 coordinates with CDK2 kinase to activate the MCM complex bound at the pre-RC, resulting in the recruitment of DNA polymerases and initiation of DNA replication.^52^ Downregulation of CDC7 as well as CDK2 and MCM gene family is associated with the inhibitory effects of genistein and TSA on DNA replication.^53^ We found that miR-630-inhibited A549 cell proliferation (Figure 4d) was associated with the downregulation of CDC7 and phospho-MCM2 (Figure 4a), which led to the blockage of CDC7-mediated initiation of DNA synthesis (Figures 4b and c). This can at least partly explain CIS-induced inhibitory proliferation. Depletion of CDC7 in cancer cells impairs progression through the S phase.^1,11,15,16^ Transfection of pre-miR-630 arrests A549 at G0/G1 by p27Kip1 induction upon CIS exposure.^36^ Similarly, we found that miR-630 transfection arrested A549 at the G1 phase(Figure 6e), and CIS exposure induced G1 arrest (Figures 6a and b), followed by miR-630 induction (Figure 6c). As miR-630 overexpression could partly diminish CIS-induced p53 (Figure 5i) due to targeting EP300 that acetylates p53,^39,40^ we suppose that the signal pathway in CIS-induced G1 arrest should differ from miR-630 transfection-resulted G1 arrest. The former depends upon ATM/ATR-p53-p21 signal pathway in A549 (Figure 6d), whereas the latter is attributed to miR-630-mediated CDC7 inactivation, which leads to defective G1–S transition.

**Figure 7:**
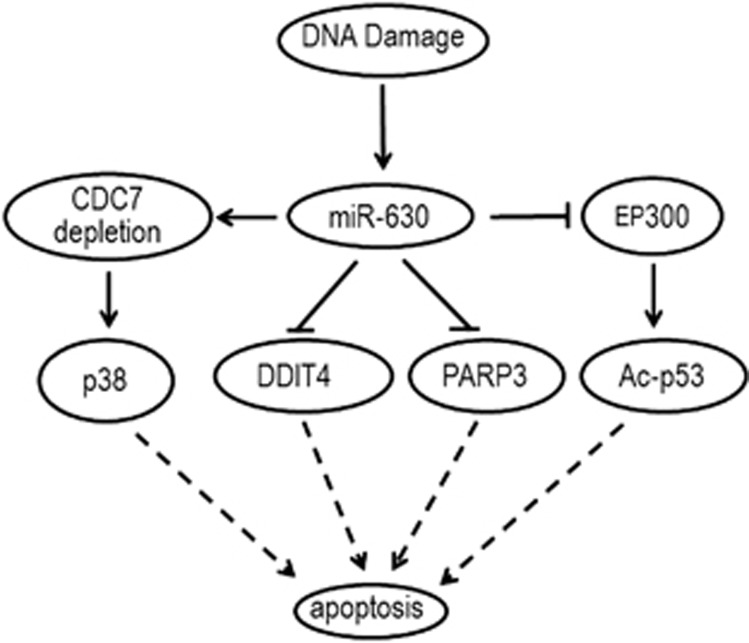
Schematic diagram showing multiple target roles of miR-630 in regulating apoptosis under DNA damage stress. MiR-630 promotes apoptosis by suppressing CDC7 expression and reduces apoptosis by direct and indirect suppressing apoptotic regulators DDIT4, PARP3, EP300 and p53

We found that silencing CDC7 caused apoptosis in A549 (Figures 3e and a, lower panel), as CIS did so (Figures 3a–c), whereas CDC7 overexpression diminished CIS-induced apoptosis (Figures 3h and i and Supplementary Figure S5), indicating that CDC7 downregulation is responsible at least partly for CIS-induced apoptosis. Surprisingly, we could not detect apoptosis in miR-630 transfected cells (Figure 5a and Supplementary Figure S6), indicating that overexpression of miR-630 and depletion of CDC7 by RNA interference (RNAi) (or downregulation of CDC7 by CIS) do not have equal effect on apoptosis, probably because of the multiple target effects of miR-630. We showed that DDIT4, PARP3 and EP300, which have been shown to be apoptotic activators,^34,38, 39, 40^ were specific targets for miR-630 (Figure 5c). MiR-630 transfection significantly reduced expression of DDIT4, PARP3 and EP300 mRNAs and proteins in A549 (Figures 5d and e). Importantly, individual silencing, in particular, combined silencing of DDIT4, PARP3 and EP300, significantly inhibited apoptosis (Figures 5f and g and Supplementary Figure S8) and procaspase-3 activation (Figure 5h). Furthermore, miR-630 overexpression markedly decreased acetylated and phosphorylated p53 and total p53 in CIS-exposed A549 (Figure 5i). EP300 may act as a regulator of p53 via its acetylase.^39,40^ Therefore, decreased acetylation of p53 should be attributed to miR-630-targeted EP300. Our data suggest that miR-630 may directly or indirectly downregulate both apoptotic activator(s) and inhibitor(s) that have a bimodal role in the regulation of apoptosis. On the one hand, miR-630 promoted apoptosis by downregulating CDC7; on the other hand, it reduced apoptosis by downregulating apoptotic activators DDIT4, PARP3, EP300 and p53, thereby maintaining apoptotic balance in DDR (Figure 7). This can explain why we could not detect apoptosis in miR-630-transfected cells (Figure 5a). Although miR-630 transfection or induction may partly offset p53 acetylation and phosphorylation, the levels of chemomodified p53 in CIS-exposed cells were still higher than control (Figure 5i). We found recently that p53 was accumulated in HCT116p53^+/+^ and A549 upon CIS-induced DNA damage.^54^ Our recent observation^54^ and the data fromFigure 5i suggest that apoptotic mechanism still works in the presence of CIS, although miR-630 downregulates p53 by targeting EP300. Notably, miR-630 overexpression is an extreme case that differs from miR-630 induction by CIS. Therefore, the bimodal role of miR-630 does not discriminate against the contributions of CDC7 inactivation and other apoptotic regulators to CIS-induced apoptosis.

In summary, we have demonstrated that miR-630 targets CDC7, thereby inhibiting CDC7-mediated initiation of DNA synthesis and inducing G1 arrest, but has a bimodal role in the regulation of apoptosis because of its multiple target effects, maintaining apoptotic balance under genotoxic stress. These data afford a comprehensive view of miRNA-dependent regulation of gene expression in regulating apoptosis in DDR.

## Materials and Methods

### Cell culture and drug treatment

Human lung cancer A549 and H1299 cells and human cervical adenocarcinoma HeLa cells were from American Type Culture collection (ATCC, Manassas, VA, USA). Human breast cancer MCF7 and MDA-MB-231 cells were a gift from Dr Yong-Feng Shang (Peking University Health Science Center, Beijing, China). Cells were cultured in DMEM medium supplemented with 10% fetal bovine serum (GIBCO-BRL, Carlsbad, CA, USA), 100 IU/ml penicillin and 100 mg/ml streptomycin, and grown at 37 °C with 5% CO_2_. CIS (100 *μ*M) (Sigma, St Louis, MO, USA), CPT (1 *μ*M) (Sigma) and Cdcl2 (50 *μ*M) (Sigma) in DMSO were added to cultures for given hours, respectively.

### Constructs, miRNAs, siRNAs and transfection

The pEGFP-C2-CDC7 and pcDNA3.1-Flag-CDC7 constructs were kindly provided by Dr Peter Cherepanov (the University of Imperial College London, St Mary's Campus, London, UK); pcDNA3.1-p53 was provided by Dr Yong-Feng Shang (Peking University Health Science Center). The sequence of human CDC7 3'-UTR was amplified using PCR and inserted into the pEGFP-C3 vector at *Kpn*I and *Bam*HI sites to generate the pEGFP-C3-CDC7-3'-UTR sensor expression plasmid. The primers for CDC73'-UTR amplification were: (forward) 5′-GGGGTACCGTAATGGATCTTCATTTAATGT-3′ and (reverse) 5′-CGGGATCCTAAAAAATATAAAAGGATAACT-3′. Synthesized miRNA mimics and siRNAs were from GenePharma (Shanghai, China), miRNA inhibitors were from Life Technologies (Waltham, MA, USA). The targeted siRNA sequences were as follows: siCDC7-1, 5′-AAGCAGUCAAAGACUGUGGAU-3′^11,15,55^ siCDC7-2, 5′-GCTCAGCAGGAAAGGTGTTTT-3′^11,55^ siDDIT4, 5′-GTGGAGACTAGAGGCAGGAGC-3′^41^ siEP300, 5′-CCCTGGATTAAGTTTGATAAA-3′^42^ siPARP3, 5′-GAGAAGAAATTTCGGGAAA-3′ (Boehler *et al.*, Supporting information, http: //www.pnas.org/cgi/content/short/1016574108).

Transfection was performed with Lipofectamine 2000 (Invitrogen, Carlsbad, CA, USA), following the manufacturer's instructions. Cells were plated in 6-well plates at 50% confluence and transfected with the miRNA mimics, miRNA inhibitors and siRNAs at final concentrations of 50 nM, and collected 48 h after transfection for further analysis.

### RNA isolation and RT-qPCR

Total RNA was isolated using the Trizol (Invitrogen) according to the manufacturer's instructions. For mRNA detection, reverse transcription was performed according to the protocol of RevertAidTM First Strand cDNA Synthesis Kit (Thermo Scientific, Waltham, MA, USA). For the analysis of mature miRNAs, 2 *μ*g total RNA was reverse transcribed using Reverse Transcription Kit (Thermo Scientific); Stem-loop RT primers for mature miRNAs and oligo(dT) were used for normal reverse transcription. RT-qPCR was performed by using the Power SYBR Green PCR Master Mix (Applied Biosystems, Carlsbad, CA, USA). RT-qPCR data were normalized to GAPDH or U6; the RT-qPCR primers used were as follows: for CDC7, (forward) 5′-AGTGCCTAACAGTGGCTGG-3′ and (reverse) 5′-CACGGTGAACAATACCAAACTGA-3′ for miR-630, 5′-ACACTCCAGCTGGGAGTATTCTGTACCAG-3′ and 5′-TGGTGTCGTGGAGTCG-3′ for GAPDH, 5′-TGTCAGTGGTGGACCTGACCT-3′ and 5′-AGGGGAGATTCAGTGTGGTG-3′ for DDIT4, 5′-TGAGGATGAACACTTGTGTGC-3′ and 5′-CCAACTGGCTAGGCATCAGC-3′ for PARP3, 5′-GCCCTGGGTACAGACTGAG-3′ and 5′-CGCTTCTCTGCGGGTATGG-3′ for EP300, 5′-AGCCAAGCGGCCTAAACTC-3′ and 5′-TCACCACCATTGGTTAGTCCC-3′ and for U6, 5′-CCTGCTTCGGCAGCACA-3′ and 5′-TGGAACGCTTCACGAA-3′ Stem-loop RT primers for miR-630, 5′-CTCAACTGGTGTCGTGGAGTCGGCAATTCAGTTGAGACCTTCCC-3′ specific RT primer for U6, 5′-AAAATATGGAACGCTTCACGAATTTGC-3′.

### Northern blotting analysis

Total RNA was extracted using Trizol reagent (Invitrogen), resolved on 12.5% denaturing polyacrylamide gel and electrotransferred to Hybond N^+^ membranes (Amersham Biosciences, Little Chalfont, UK), followed by UV crosslinking the membrane two times at 120 mJ for 30 s each. After prehybridization for 2 h at 42 °C in a highly efficient hybridized solution (Mylab, Beijing, China), the membrane was hybridized overnight with the Dig-labeled probes corresponding to miR-630 (synthesized by Sangon, Shanghai, China) in a highly efficient hybridized solution. Following hybridization, immunologic detection was performed using DIG Detection Kit (Mylab). The membrane was washed two times with 2 × SSC and 0.05% SDS at room temperature (RT) for 5 min each, two times with 0.1 × SSC and 0.1% SDS at 50 °C for 15 min each and one time with MABT (0.1 M maleic acid, 0.15 M NaCl, 0.3% Tween-20, pH 7.5) at RT for 5 min. The membrane was blocked with blocking buffer at RT for 30 min, and then hybridized overnight with Dig-AP in 1 × blocking buffer at 4 °C. After incubation with Dig-AP, blots were visualized by Enhanced Chemiluminescence Regents CDP-star (Mylab).

### Western blotting

Whole-cell extracts were prepared in lysis buffer and the concentration of proteins was determined using the BCA Protein Assay Reagent Kit (Thermo scientific). Proteins were separated in 10% SDS-PAGE and transferred to nitrocellulose membrane. The membrane was blocked using TBST buffer containing 5% (w/v) non-fat milk 1 h at RT and probed with specific antibodies (1 : 1000) for CDC7 (sc-56275, Santa Cruz Biotechnology, Santa Cruz, CA, USA), caspase-3 (active) (1476-1; Epitomics, CA, USA), p38 (Y122) (ab32142; Abcam), phospho-p38 MAPK (pT180/pY182) (1229S; Epitomics), MCM2 (CST-3619; Cell Signaling), phospho-MCM2 (Ser139) (CST-12958), PARP3(ab96601), DDIT4(ab63059), ATM (CST-2873S), ATM phospho (pS1981) (2152-1), ATR (N19) (sc-1887), phospho-ATR (Ser428) (CST-2853S), cyclin D1 (ab24249), p53 (CST-2527), phospho-p53 (Ser15) (CST-9284), phospho-p53 (Ser20) (CST-9287), phospho-p53 (Ser46) (CST-2521), Ac-p53 (Lys382) (CST-2525), *β*-actin (PM053; MBL, Nagoya, Japan) and *α*-tubulin (PM054; MBL). Blots were developed with a secondary antibody (IgG)-conjugated horseradish peroxidase. Chemiluminescence signals were visualized using SuperSignal1 West Pico Chemiluminescent Substrate (Pierce, Rockford, IL, USA).

### Luciferase reporter constructs and luciferase assay

For construction of luciferase reporter plasmids containing 3'-UTR of DDIT4, PARP3, EP300, CDC7 and its truncates (fragments A to E) or point mutants, the sequences of 3'-UTRs and CDC7 3'-UTR fragments A to E (Figure 1g, upper panel) and point mutants (Figure 1f) were obtained by PCR and inserted into the pMIR-Report plasmid (Applied Biosystems) at the *Spe*I and *Hin*dIII sites to generate pMIR-Report-PmiR-3'-UTR, pMIR-Report-PmiR-A, -B, -C, -D, -E (Figure 1g, lower panel) and fragments ‘A' to ‘E' mutants of pMIR-Report-PmiR. For reporter enzyme assays, cells were co-transfected with 100 ng of firefly luciferase reporter vector containing a wild-type or mutant target site, 5 ng of *Renilla* luciferase for normalizing transfection efficiency and 10 nM miR-630 mimics in 24-well plates using Lipofectamine 2000. Cells were collected 48 h after transfection. Luciferase assays were performed using the Dual Luciferase Reporter Assay System (Promega, Madison, WI, USA) according to the manufacturer's instructions. The primer pairs used were as follows: CDC7 full-length 3′-UTR: (F) 5′-GGACTAGTCCTAATGGATCTTCATTTAATGTTTAC-3′ and (R) 5′-CCCAAGCTTGGGTAAAAAATATAAAAGGATAACTTTATTG-3′ Fragment A: (F) 5′-GGACTAGTCCTAATGGATCTTCATTTAATGTTTAC-3′ and (R) 5′-CCCAAGCTTTTTAGAATGTGCCACCAA-3′ MuCDC7 for binding site 1: (F) 5′-GGACTAGTCCTAATGGATCTTCATTTAATGTTTACTGTTATGAGGTAG AATAAAAAAGACCTCTTTGTAATAGCCACAAG-3′ and (R) 5′-CCCAAGCTTTTTAGAATGTGCCACCAA-3′ Fragment B: (F) 5′-GGACTAGTTGTAATAGCCACAAGTTC-3′ and (R) 5′-CCCAAGCTTTAATCCTCATCACATCTG-3′ MuCDC7 for binding site 2: (F) 5′-ATTCTAAAATATAGATTAAGACCTCTTAAAATGCCTGGGAT-3′ and (R) 5′-ATCCCAGGCATTTTAAGAGGTCTTAATCTATATTTTAGAAT-3′ Fragment C: (F) 5′-GGACTAGT GTGATGAGGATTAAATGA-3′ and (R) 5′-CCCAAGCTTCAGAAACTTTGTGGTCAG-3′ Fragment D: (F) 5′-GGACTAGTAAGTTTCTGGATGTTTTA-3′ and (R) 5′-CCCAAGCTTTTGCCTACTTCATTATCT-3′ MuCDC7 for binding site 3: (F) 5′-CTGCTGAAAGGAAAAGTGACCTCAGAATTGACGGTATTAT-3′ and (R) 5′-ATAATACCGTCAATTCTGTATTCACTTTTCCTTTCAGCAG-3′ Fragment E: (F) 5′-GGACTAGTAAGTAGGCAAAGAGAAAAGG-3′ and (R) 5′-CCCAAGCTTGGGTAAAAAATATAAAAGGATAACTTTATTG-3′ MuCDC7 for binding site 4: (F) 5′-CCCATTTAGTAGTCATAGACCTCAGAAATAGTTTAGGGAC-3′ and (R) 5′-GTCCCTAAACTATTTCTGAGGTCTATGACTACTAAATGGG-3′ DDIT4 full-length 3′-UTR: (F) 5′-GGACTAGT ATTGAGGAGTGTTGAACTTCA-3′ and (R) 5′-CCCAAGCTTAACTGTTTTAACAAACATGTTTATT-3′ MuDDIT4 for binding site: (F) 5′-AGATACTCACTGTTCATGACCTCACTTGATGTTCAAGTAT-3′ and (R) 5′-ATACTTGAACATCAAGTGAGGTCATGAACAGTGAGTATCT-3′ PPRP3 full-length 3′-UTR: (F) 5′-GGACTAGTCTGGAGGTCCACCTCTGA-3′ and (R) 5′-CCCAAGCTTAAGGAGGAAATCTTGTCA-3′ MuPPRP3 for binding site: (F) 5′-TATCACTCCTTTTTTTCAAGACCTCAATACGTTGTTGTTA-3′ and (R) 5′-TAACAACAACGTATTGAGGTCTTGAAAAAAAGGAGTGATA-3′ EP300 full-length 3′-UTR: (F) 5′-GGACTAGTAGACACCTTGTAGTATTTTGG-3′ and (R) 5′-CCCAAGCTTTGTCTGTCTCACACAGTTTAT-3′ MuEP300 for binding site: (F) 5′-GAACCTGAGGGATGATAGACCTCAAAGAATATATTTTTGT-3′ and (R) 5′-ACAAAAATATATTCTTTGAGGTCTATCATCCCTCAGGTTC-3′.

### Cell survival assay

Cells were plated at 96-well plates and treated with given molecules. Cell growth was analyzed using CellTiter 96 AQueous Non-Radioactive Cell Proliferation Assay (MTS) (Promega) according to the manufacturer's instructions.

### Flow cytometry

To assay apoptosis, A549 cells were transfected with miR-630 mimic or CDC7 siRNA and harvested for analysis 48 h after transfection by trypsinization, and then stained with Annexin V/PI Apoptosis Detection Kit (Biosea, Beijing, China) according to manufacturer's instructions. Cell apoptosis were analyzed with a FACScalibur flow cytometer (BD Biosciences, San Jose, CA, USA). The fluorescence signals of apoptosis cells were represented by Annexin^+^/PI^−^ and Annexin^+^/PI^+^. For BrdU incorporation, cells were labeled with 50 *μ*M BrdU for 1 h. Cells were washed in PBS, fixed in ice-cold 70% ethanol for 24 h and later DNA was denatured with 2 N HCl for 30 min. Incorporated BrdU was detected using FITC-conjugated anti-BrdU antibody (11-5071; eBioscience, San Diego, CA, USA).

### Statistical analysis

The Student's *t*-test and Wilcoxon's rank-sum test were used for statistical analysis. Statistical significance was defined by a two-tailed *P*-value of 0.05.

## Abbreviations

CDC7: cell-cycle kinase 7
miRNA: microRNA
miR-630: microRNA-630
CIS: cisplatin
CPT: camptothecin
RT-qPCR: real-time quantitative PCR
PI: propidium iodide
BrdU: bromo-deoxyuridine
RNAi: RNA interference
DDR: DNA damage response

## References

[bib1] Johnston LH Masai H Sugino A First the CDKs, now the DDKs Trends Cell Biol 1999 9 249 252 10370238 10.1016/s0962-8924(99)01586-x

[bib2] Sclafani RA Cdc7p-Dbf4p becomes famous in the cell cycle J Cell Sci 2000 113 (Part 12 2111 2117 10825284 10.1242/jcs.113.12.2111

[bib3] Hughes S Elustondo F Di Fonzo A Leroux FG Wong AC Snijders AP Crystal structure of human CDC7 kinase in complex with its activator DBF4 Nat Struct Mol Biol 2012 19 1101 1107 23064647 10.1038/nsmb.2404

[bib4] Montagnoli A Bosotti R Villa F Rialland M Brotherton D Mercurio C Drf1, a novel regulatory subunit for human Cdc7 kinase EMBO J 2002 21 3171 3181 12065429 10.1093/emboj/cdf290 PMC126049

[bib5] Dowell SJ Romanowski P Diffley JF Interaction of Dbf4, the Cdc7 protein kinase regulatory subunit, with yeast replication origins *in vivo* Science 1994 265 1243 1246 8066465 10.1126/science.8066465

[bib6] Kumagai H Sato N Yamada M Mahony D Seghezzi W Lees E A novel growth- and cell cycle-regulated protein, ASK, activates human Cdc7-related kinase and is essential for G1/S transition in mammalian cells Mol Cell Biol 1999 19 5083 5095 10373557 10.1128/mcb.19.7.5083 PMC84351

[bib7] Jiang W McDonald D Hope TJ Hunter T Mammalian Cdc7-Dbf4 protein kinase complex is essential for initiation of DNA replication EMBO J 1999 18 5703 5713 10523313 10.1093/emboj/18.20.5703 PMC1171637

[bib8] Bartek J Lukas C Lukas J Checking on DNA damage in S phase Nat Rev Mol Cell Biol 2004 5 792 804 15459660 10.1038/nrm1493

[bib9] Jares P Donaldson A Blow JJ The Cdc7/Dbf4 protein kinase: target of the S phase checkpoint EMBO Rep 2000 1 319 322 11269496 10.1093/embo-reports/kvd076 PMC1083750

[bib10] Aguilera A Gomez-Gonzalez B Genome instability: a mechanistic view of its causes and consequences Nat Rev Genet 2008 9 204 217 18227811 10.1038/nrg2268

[bib11] Tsuji T Lau E Chiang GG Jiang W The role of Dbf4/Drf1-dependent kinase Cdc7 in DNA-damage checkpoint control Mol Cell 2008 32 862 869 19111665 10.1016/j.molcel.2008.12.005 PMC4556649

[bib12] Kim JM Kakusho N Yamada M Kanoh Y Takemoto N Masai H Cdc7 kinase mediates Claspin phosphorylation in DNA replication checkpoint Oncogene 2008 27 3475 3482 18084324 10.1038/sj.onc.1210994

[bib13] Bonte D Lindvall C Liu HY Dykema K Furge K Weinreich M Cdc7-Dbf4 kinase overexpression in multiple cancers and tumor cell lines is correlated with p53 inactivation Neoplasia 2008 10 920 931 18714392 10.1593/neo.08216 PMC2517636

[bib14] Hess GFDR Weiland KL Slightom JL Sclafani RA Hollingsworth RE A human homolog of the yeast CDC7 gene is overexpressed in some tumors and transformed cell lines Gene 1998 211 133 140 9573348 10.1016/s0378-1119(98)00094-8

[bib15] Montagnoli A Tenca P Sola F Carpani D Brotherton D Albanese C Cdc7 inhibition reveals a p53-dependent replication checkpoint that is defective in cancer cells Cancer Res 2004 64 7110 7116 15466207 10.1158/0008-5472.CAN-04-1547

[bib16] Im JS Lee JK ATR-dependent activation of p38 MAP kinase is responsible for apoptotic cell death in cells depleted of Cdc7 J Biol Chem 2008 283 25171 25177 18625709 10.1074/jbc.M802851200

[bib17] Montagnoli A Moll J Colotta F Targeting cell division cycle 7 kinase: a new approach for cancer therapy Clin Cancer Res 2010 16 4503 4508 20647475 10.1158/1078-0432.CCR-10-0185

[bib18] Swords R Mahalingam D O'Dwyer M Santocanale C Kelly K Carew J Cdc7 kinase – a new target for drug development Eur J Cancer 2010 46 33 40 19815406 10.1016/j.ejca.2009.09.020

[bib19] Lewis BP Burge CB Bartel DP Conserved seed pairing, often flanked by adenosines, indicates that thousands of human genes are microRNA targets Cell 2005 120 15 20 15652477 10.1016/j.cell.2004.12.035

[bib20] Croce CM Calin GA miRNAs, cancer, and stem cell division Cell 2005 122 6 7 16009126 10.1016/j.cell.2005.06.036

[bib21] Calin GA Croce CM MicroRNA signatures in human cancers Nat Rev Cancer 2006 6 857 866 17060945 10.1038/nrc1997

[bib22] Esquela-Kerscher A Slack FJ Oncomirs – microRNAs with a role in cancer Nat Rev Cancer 2006 6 259 269 16557279 10.1038/nrc1840

[bib23] Yanaihara N Caplen N Bowman E Seike M Kumamoto K Yi M Unique microRNA molecular profiles in lung cancer diagnosis and prognosis Cancer Cell 2006 9 189 198 16530703 10.1016/j.ccr.2006.01.025

[bib24] Calin GA Ferracin M Cimmino A Di Leva G Shimizu M Wojcik SE A micro-RNA signature associated with prognosis and progression in chronic lymphocytic leukemia N Engl J Med 2005 353 1793 1801 16251535 10.1056/NEJMoa050995

[bib25] Kong YW Ferland-McCollough D Jackson TJ Bushell M MicroRNAs in cancer management Lancet Oncol 2012 13 e249 e258 22652233 10.1016/S1470-2045(12)70073-6

[bib26] Chang TC Wentzel EA Kent OA Ramachandran K Mullendore M Lee KH Transactivation of miR-34a by p53 broadly influences gene expression and promotes apoptosis Mol Cell 2007 26 745 752 17540599 10.1016/j.molcel.2007.05.010 PMC1939978

[bib27] He L He X Lim LP de Stanchina E Xuan Z Liang Y A microRNA component of the p53 tumour suppressor network Nature 2007 447 1130 1134 17554337 10.1038/nature05939 PMC4590999

[bib28] Raver-Shapira N Marciano E Meiri E Spector Y Rosenfeld N Moskovits N Transcriptional activation of miR-34a contributes to p53-mediated apoptosis Mol Cell 2007 26 731 743 17540598 10.1016/j.molcel.2007.05.017

[bib29] Cannell IG Kong YW Johnston SJ Chen ML Collins HM Dobbyn HC P38 MAPK/MK2-mediated induction of miR-34c following DNA damage prevents Myc-dependent DNA replication Proc Natl Acad Sci USA 2010 107 5375 5380 20212154 10.1073/pnas.0910015107 PMC2851793

[bib30] Lal A Pan Y Navarro F Dykxhoorn DM Moreau L Meire E MiR-24-mediated downregulation of H2AX suppresses DNA repair in terminally differentiated blood cells Nat Struct Mol Biol 2009 16 492 498 19377482 10.1038/nsmb.1589 PMC2853019

[bib31] Pothof J Verkaik NS van IW Wiemer EA Ta VT van der Horst GT MicroRNA-mediated gene silencing modulates the UV-induced DNA-damage response EMBO J 2009 28 2090 2099 19536137 10.1038/emboj.2009.156 PMC2718280

[bib32] Hu H Du L Nagabayashi G Seeger RC Gatti RA ATM is down-regulated by N-Myc-regulated microRNA-421 Proc Natl Acad Sci USA 2010 107 1506 1511 20080624 10.1073/pnas.0907763107 PMC2824372

[bib33] Wang Y Huang JW Li M Cavenee WK Mitchell PS Zhou X MicroRNA-138 modulates DNA damage response by repressing histone H2AX expression Mol Cancer Res 2011 9 1100 1111 21693595 10.1158/1541-7786.MCR-11-0007 PMC3157593

[bib34] Huang Y Chuang A Hao H Talbot C Sen T Trink B Phospho-DeltaNp63alpha is a key regulator of the cisplatin-induced microRNAome in cancer cells Cell Death Differ 2011 18 1220 1230 21274007 10.1038/cdd.2010.188 PMC3131951

[bib35] Farhana L Dawson MI Murshed F Das JK Rishi AK Fontana JA Upregulation of miR-150* and miR-630 induces apoptosis in pancreatic cancer cells by targeting IGF-1R PLoS One 2013 8 e61015 23675407 10.1371/journal.pone.0061015 PMC3651232

[bib36] Galluzzi L Morselli E Vitale I Kepp O Senovilla L Criollo A miR-181a and miR-630 regulate cisplatin-induced cancer cell death Cancer Res 2010 70 1793 1803 20145152 10.1158/0008-5472.CAN-09-3112

[bib37] Landgraf P Rusu M Sheridan R Sewer A Iovino N Aravin A A mammalian microRNA expression atlas based on small RNA library sequencing Cell 2007 129 1401 1414 17604727 10.1016/j.cell.2007.04.040 PMC2681231

[bib38] Knowles LM Yang C Osterman A Smith JW Inhibition of fatty-acid synthase induces caspase-8-mediated tumor cell apoptosis by up-regulating DDIT4 J Biol Chem 2008 283 31378 31384 18796435 10.1074/jbc.M803384200 PMC2581575

[bib39] Grossman SR Perez M Kung AL Joseph M Mansur C Xiao ZX p300/MDM2 complexes participate in MDM2-mediated p53 degradation Mol Cell 1998 2 405 415 9809062 10.1016/s1097-2765(00)80140-9

[bib40] Shi D Pop MS Kulikov R Love IM Kung AL Grossman SR CBP and p300 are cytoplasmic E4 polyubiquitin ligases for p53 Proc Natl Acad Sci USA 2009 106 16275 16280 19805293 10.1073/pnas.0904305106 PMC2752525

[bib41] Song S Abdelmohsen K Zhang Y Becker KG Gorospe M Bernier M Impact of pyrrolidine dithiocarbamate and interleukin-6 on mammalian target of rapamycin complex 1 regulation and global protein translation J Pharmacol Exp Ther 2011 339 905 913 21917559 10.1124/jpet.111.185678 PMC3226362

[bib42] Chen W Jia W Wang K Si X Zhu S Duan T Kang J Distinct roles for CBP and p300 on the RA-mediated expression of the meiosis commitment gene Stra8 in mouse embryonic stem cells PLoS One 2013 8 e66076 23785470 10.1371/journal.pone.0066076 PMC3681938

[bib43] Kurita M Suzuki H Masai H Mizumoto K Ogata E Nishimoto I Overexpression of CR/periphilin downregulates Cdc7 expression and induces S-phase arrest Biochem Biophys Res Commun 2004 324 554 561 15474462 10.1016/j.bbrc.2004.09.083

[bib44] Barkley LR Santocanale C MicroRNA-29a regulates the benzo[*a*]pyrene dihydrodiol epoxide-induced DNA damage response through Cdc7 kinase in lung cancer cells Oncogenesis 2013 2 e57 23877787 10.1038/oncsis.2013.20 PMC3740286

[bib45] O'Donnell KA Wentzel EA Zeller KI Dang CV Mendell JT c-Myc-regulated microRNAs modulate E2F1 expression Nature 2005 435 839 843 15944709 10.1038/nature03677

[bib46] Tarasov V Jung P Verdoodt B Lodygin D Epanchintsev A Menssen A Differential regulation of microRNAs by p53 revealed by massively parallel sequencing: miR-34a is a p53 target that induces apoptosis and G1-arrest Cell Cycle 2007 6 1586 1593 17554199 10.4161/cc.6.13.4436

[bib47] Woods K Thomson JM Hammond SM Direct regulation of an oncogenic micro-RNA cluster by E2F transcription factors J Biol Chem 2007 282 2130 2134 17135268 10.1074/jbc.C600252200

[bib48] Petrocca F Visone R Onelli MR Shah MH Nicoloso MS de Martino I E2F1-regulated microRNAs impair TGFbeta-dependent cell-cycle arrest and apoptosis in gastric cancer Cancer Cell 2008 13 272 286 18328430 10.1016/j.ccr.2008.02.013

[bib49] Lize M Pilarski S Dobbelstein M E2F1-inducible microRNA 449a/b suppresses cell proliferation and promotes apoptosis Cell Death Differ 2010 17 452 458 19960022 10.1038/cdd.2009.188

[bib50] Hu H Gatti RA MicroRNAs: new players in the DNA damage response J Mol Cell Biol 2011 3 151 158 21183529 10.1093/jmcb/mjq042 PMC3104011

[bib51] Cao J-X Li S-Y An G-S Mao Z-B Jia H-T Ni J-H E2F1-regulated DROSHA promotes miR-630 biosynthesis in cisplatin-exposed cancer cells Biochem Biophys Res Commun 2014 450 470 475 24909689 10.1016/j.bbrc.2014.05.138

[bib52] Sheu YJ Stillman B Cdc7-Dbf4 phosphorylates MCM proteins via a docking site-mediated mechanism to promote S phase progression Mol Cell 2006 24 101 113 17018296 10.1016/j.molcel.2006.07.033 PMC2923825

[bib53] Majid S Dar AA Saini S Chen Y Shahryari V Liu J Regulation of minichromosome maintenance gene family by microRNA-1296 and genistein in prostate cancer Cancer Res 2010 70 2809 2818 20332239 10.1158/0008-5472.CAN-09-4176

[bib54] Zhou Z Cao J-X Li S-Y An G-S Ni J-H Jia H-T P53 suppresses E2F1-dependent PLK1 expression upon DNA damage by forming p53–E2F1–DNA complex Exp Cell Res 2013 319 3104 3115 24076372 10.1016/j.yexcr.2013.09.012

[bib55] Masai H Taniyama C Ogino K Matsui E Kakusho N Matsumoto S Phosphorylation of MCM4 by Cdc7 kinase facilitates its interaction with Cdc45 on the chromatin J Biol Chem 2006 281 39249 39261 17046832 10.1074/jbc.M608935200

