# Supplementary figures and images for: MiR-630 inhibits proliferation by targeting CDC7 kinase, but maintains the apoptotic balance by targeting multiple modulators in human lung cancer A549 cells
Source: Cell Death Dis. 2014 Sep 25;5(9):e1426–. doi: 10.1038/cddis.2014.386 (PMC4225225; doi:10.1038/cddis.2014.386)

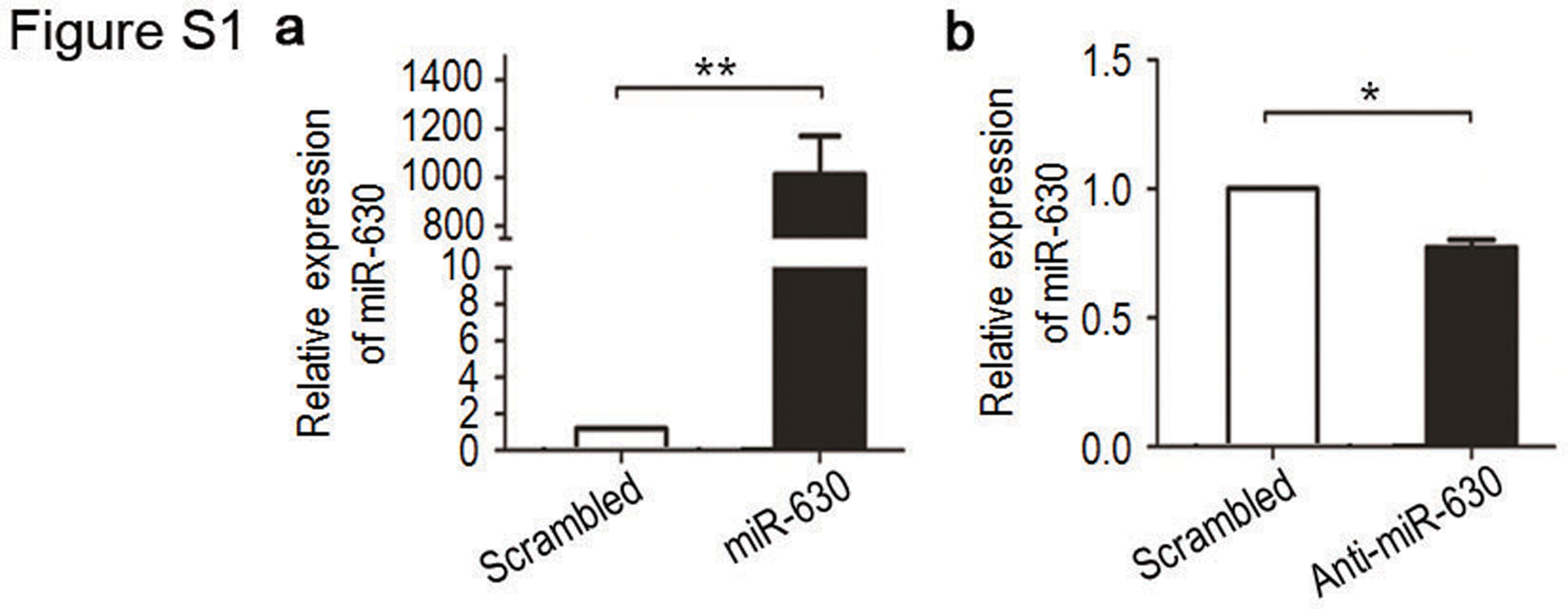

Supplement: Supplementary Figure S1 [file cddis2014386x1.tif]

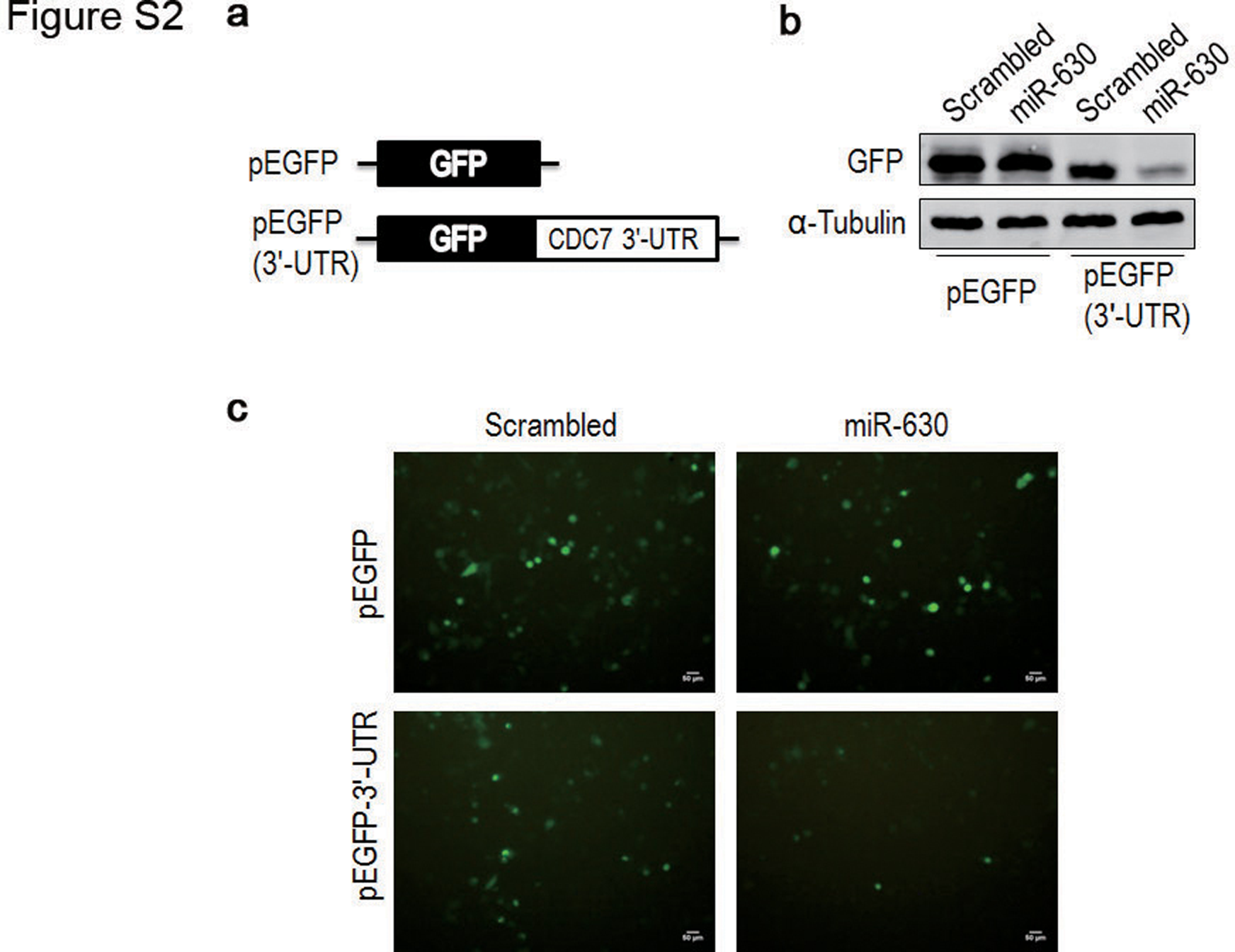

Supplement: Supplementary Figure S2 [file cddis2014386x2.tif]

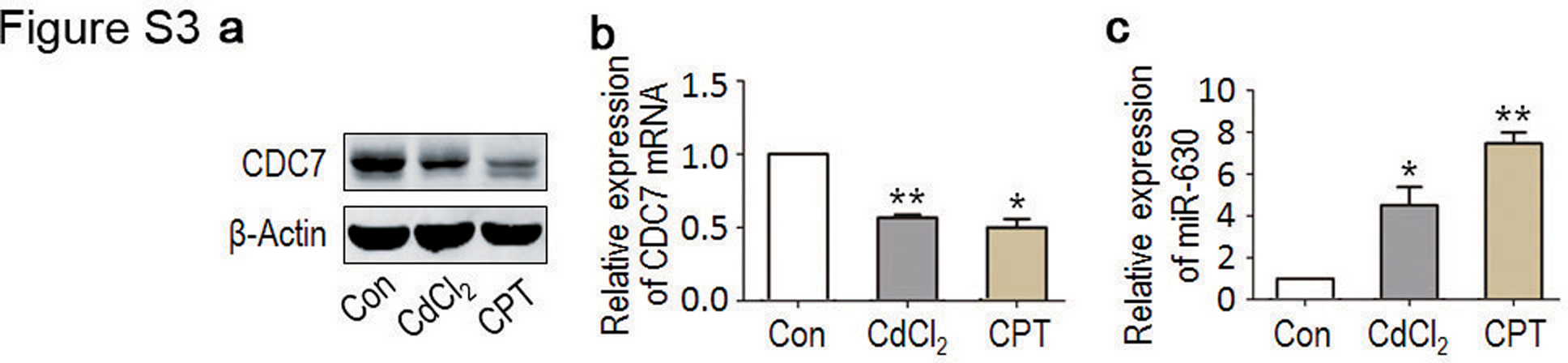

Supplement: Supplementary Figure S3 [file cddis2014386x3.tif]

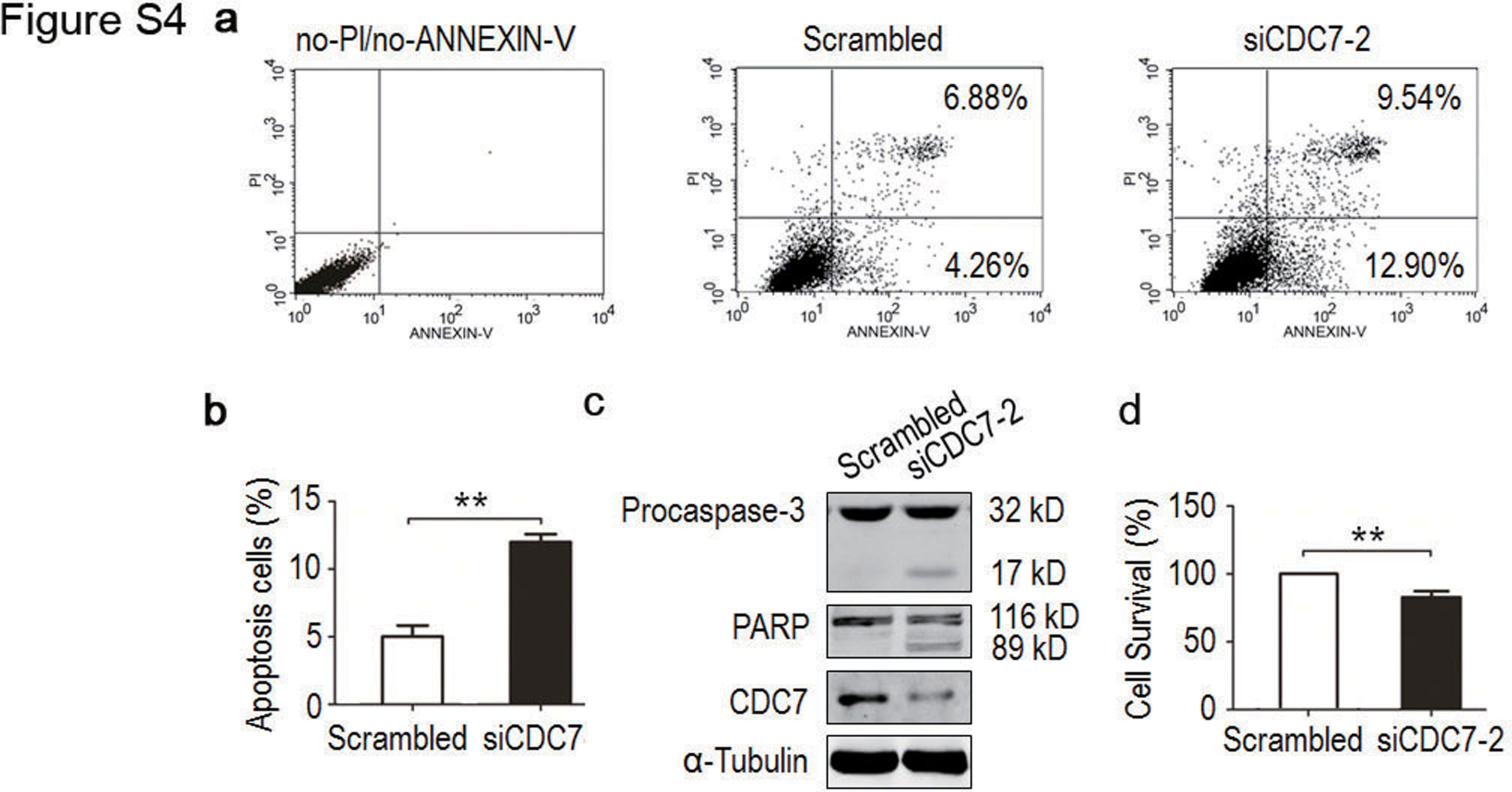

Supplement: Supplementary Figure S4 [file cddis2014386x4.tif]

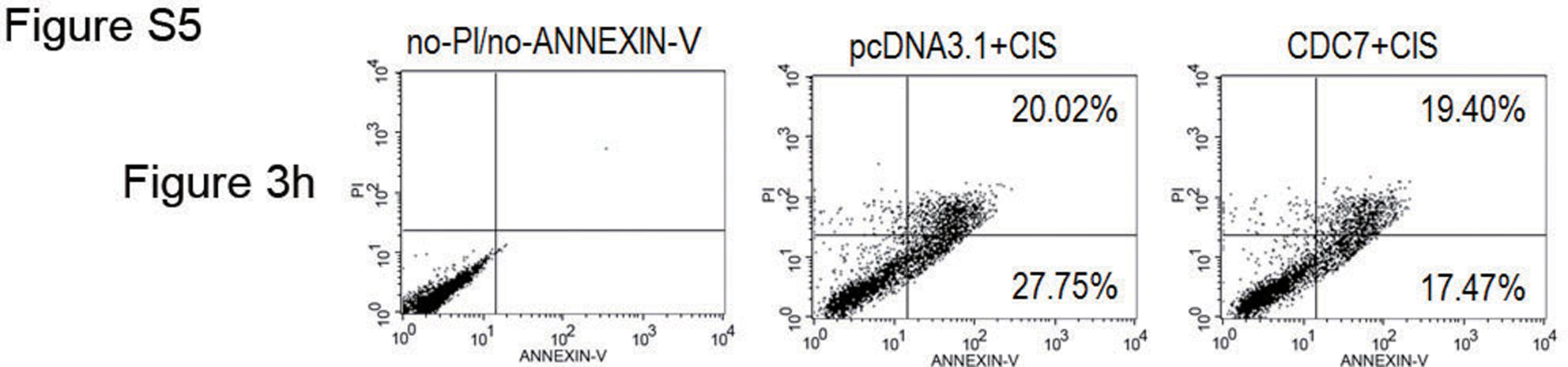

Supplement: Supplementary Figure S5 [file cddis2014386x5.tif]

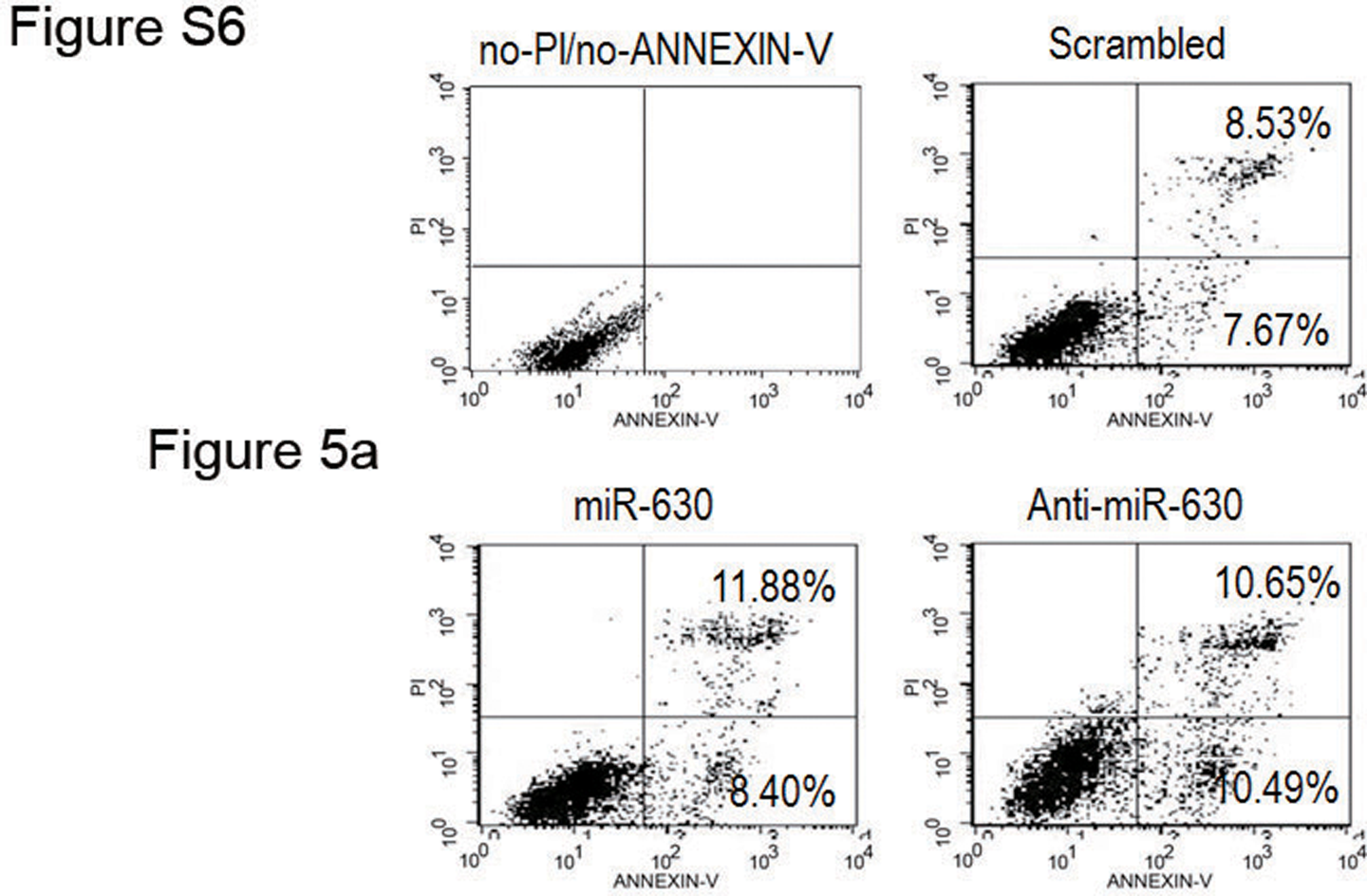

Supplement: Supplementary Figure S6 [file cddis2014386x6.tif]

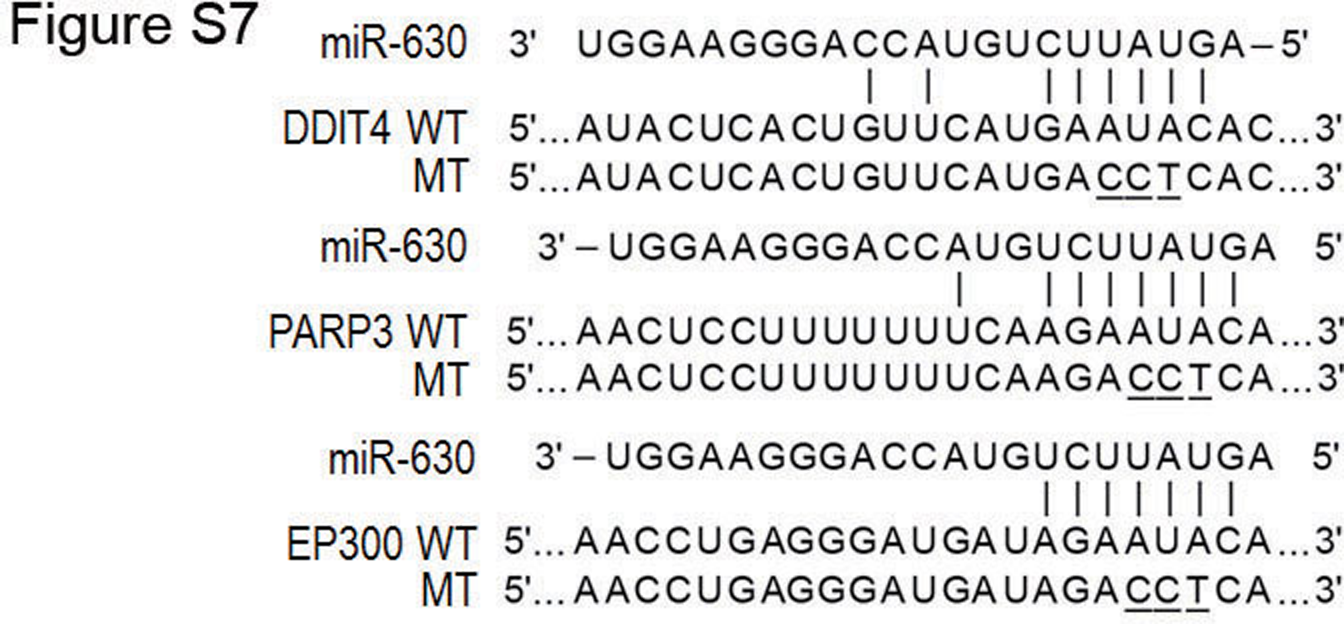

Supplement: Supplementary Figure S7 [file cddis2014386x7.tif]

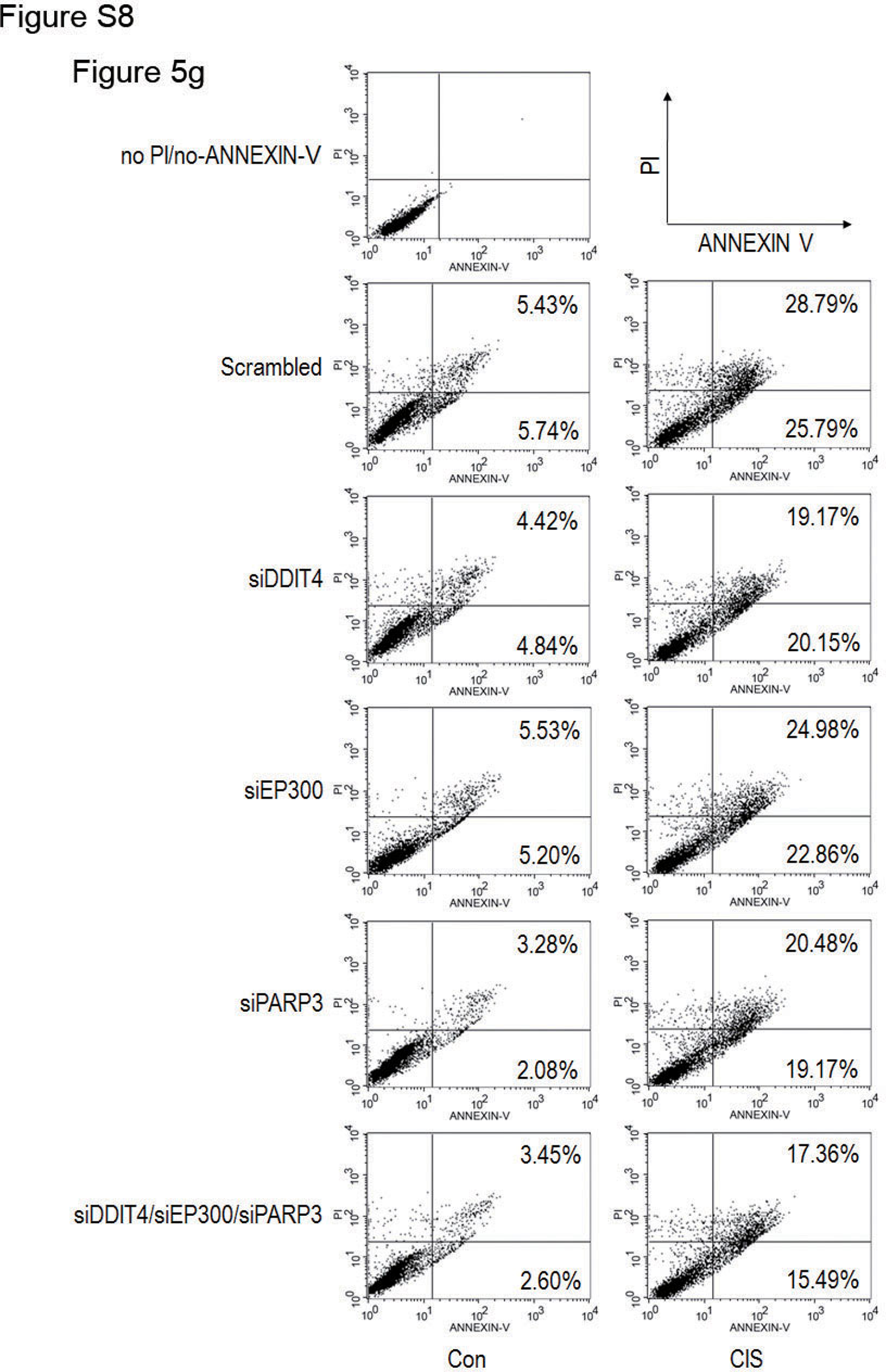

Supplement: Supplementary Figure S8 [file cddis2014386x8.tif]
