# Supplementary material for: MiR-630 inhibits proliferation by targeting CDC7 kinase, but maintains the apoptotic balance by targeting multiple modulators in human lung cancer A549 cells
Source: Cell Death Dis. 2014 Sep 25;5(9):e1426–. doi: 10.1038/cddis.2014.386 (PMC4225225; doi:10.1038/cddis.2014.386)
Supplement: Supplementary Figure Legends [file cddis2014386x9.doc]

**SUPPLEMENTARY TITLES AND LEGENDS TO FIGURES**

**Supplementary Figure S1 The transfection efficiency of miR-630 mimic and inhibitor in A549 cells.** Cells were transfected with scrambled siRNA or miR-630 mimic (**a**), anti-scrambled siRNA or anti-miR-630 (**b**). The levels of miR-630 were determined by RT-qPCR. Data present mean ± SD (n=3~5). **p*<0.05; ***p*<0.01.

**Supplementary Figure S2 MiR-630 down-regulates GFP protein whose mRNA fused with CDC7 3'-UTR.** (**a**) Interpretation of plasmid pEGFP-3'-UTR. (**b**) Western blotting for reporter GFP protein after transfection of pEGFP or pEGFP-3'-UTR into A549 for 48 h. (**c**) A549 cells were photogarphed 48 h after co-transfection of scrambled siRNA or miR-630 mimic with pEGFP or pEGFP-3'-UTR plasmid, using inverted flurescencemicroscopy. Scale bar, 50 μm.

**Supplementary Figure S3 MiR-630 expression inversely correlates with CDC7 under CPT and CdCl_2_ exposure.** A549 cells were exposed to CPT (1 μM) and CdCl_2_ (50 μM) for 36 h. (**a**) Western blotting for analyzing CDC7 protein. (**b**) and (**c**) CDC7 mRNA and miR-630 expression was analyzes by RT-qPCR. Data present mean ± SD (n=3~5). **p*<0.05; ***p*<0.01.

**Supplementary Figure S4 Silencing CDC7 induces inhibitory proliferation and apoptosis in A549 cells.** (**a**) Representative dot plots of siCDC7-2 induced apoptosis. (**b**) and (**c**) Induction of apoptosis by silencing CDC7. A549 cells were transfected with scrambled siRNA or CDC7 siRNA-2 for 48 h, followed by Western blotting for activated caspase-3 and CDC7 (**c**) and by flow cytometry (**b**). Data present mean ± SD (n=4); ***p*=0.0022. (**d**) MTS assay for the survival of CDC7-silenced A549. Data present mean ± SD (n=3); ***p*=0.0053.

**Supplementary Figure S5 Flow cytometry analysis of apoptosis.** Figure 3h, representative dot plots for reduction of CIS-induced apoptosis by CDC7 over-expression.

**Supplementary Figure S6 Flow cytometry analysis of apoptosis in Figure 5a**. Representative dot plots of A549 cells transfected with scrambled siRNA, miR-630 mimic and miR-630 inhibitor for 48 h.

**Supplementary Figure S7** The predicted miR-630–binding sequences or mutated versions of DDIT4, PARP3 and EP300 3'-UTR. WT, wild type; MT, mutant (mutated bases are underlined).

**Supplementary Figure S8 Flow cytometry analysis of apoptosis in Figure 5g.** Representative dot plots of A549 cells, which were transfected with scrambled siRNA (control) or specific siRNA oligonucleotides to silence DDIT4, EP300 and PARP3 for 48 h.
